# Evolution, and functional analysis of Natural Resistance-Associated Macrophage Proteins (NRAMPs) from *Theobroma cacao* and their role in cadmium accumulation

**DOI:** 10.1038/s41598-018-32819-y

**Published:** 2018-09-26

**Authors:** Ihsan Ullah, Yirong Wang, David J. Eide, Jim M. Dunwell

**Affiliations:** 1 School of Agriculture, Policy and Development, University of Reading, Earley Gate, Reading, RG6 6AR UK; 2 Department of Nutritional Sciences, University of Wisconsin-Madison, Madison, WI 53706 USA

## Abstract

The presence of the toxic metal cadmium (Cd^2+^) in certain foodstuffs is recognised as a global problem, and there is increasing legislative pressure to reduce the content of Cd in food. The present study was conducted on cacao (*Theobroma cacao*), the source of chocolate, and one of the crops known to accumulate Cd in certain conditions. There are a range of possible genetic and agronomic methods being tested as a route to such reduction. As part of a gene-based approach, we focused on the Natural Resistance-Associated Macrophage Proteins (NRAMPS), a family of proton/metal transporter proteins that are evolutionarily conserved across all species from bacteria to humans. The plant *NRAMP* gene family are of particular importance as they are responsible for uptake of the nutritionally vital divalent cations Fe^2+^, Mn^2+^, Zn^2+^, as well as Cd^2+^. We identified the five *NRAMP* genes in cacao, sequenced these genes and studied their expression in various organs. We then confirmed the expression patterns in response to variation in nutrient cation availability and addition of Cd^2+^. Functional analysis by expression in yeast provided evidence that *NRAMP5* encoded a protein capable of Cd^2+^ transport, and suggested this gene as a target for genetic selection/modification.

## Introduction

Natural Resistance-Associated Macrophage Proteins (NRAMPS) represent a family of proton/metal transporter proteins that are evolutionarily conserved across all species from bacteria to humans^1,2^. The plant NRAMP gene family are of particular importance as they are responsible for uptake of the nutritionally vital divalent cations Fe^2+^, Mn^2+^, Zn^2+^, and Cd^2+^ a toxic metal with no known role in plant growth and development. Cadmium (Cd^2+^) is present in the agricultural environment from either geological sources, industrial pollution, or from the application of rock phosphate fertiliser, which can contain relatively high levels of Cd^2+^. Humans absorb plant-derived Cd^2+^ either from their diet, or from smoking, and it accumulates in the body during life. Because of the known ill effects of Cd^2+^ there has been much global regulatory pressure in recent years to reduce human exposure, and this has focused attention on those food ingredients, including cereals and cereal products, vegetables, nuts and pulses, starchy roots or potatoes, and meat and meat products, which are the main source of Cd^2+^ in the diet. Of specific relevance is the decision of the EU regulatory authorities to reduce from 2019 the permissible levels of dietary Cd^2+^ (Commission Regulation (EU) no 488/2014). In an attempt to reduce the levels of Cd^2+^ in crops, including cacao (the source of chocolate), a variety of research studies are underway to develop agronomic^3,4^ or genetic^5^ solutions to this problem.

This present study focused on the characterization of the NRAMP gene family in cacao and was designed to investigate the possible role of these proteins in controlling levels of Cd^2+^ in this crop. It involved an initial survey of the gene family across all plants and then the isolation, study of expression, and functional characterization of selected members of the family shown to be present in the cacao genome. It is hoped that such investigations will inform genetic approaches to selecting cacao clones with reduced Cd^2+^ or using gene-editing methods to modify existing clones.

## Results

### Overview of NRAMP gene family in selected viridiplantae species

#### Prediction of homologs

A BlastP search was conducted using the *Arabidopsis* metal transporters NRAMP1 to 6 protein sequences as queries to identify orthologs in two algae and 28 other plant species (http://www.phytozome.net/). After deletion of repetitive and short coverage sequences, a total of 170 sequences were selected. Retrieved protein sequences included two sequences each for the algal species *Chlamydomonas reinhardtii* and *Volvox carteri*. The moss species *Physcomitrella patens* and *Sphagnum fallax* had three and four NRAMPs, respectively. Among the higher plants, six NRAMP homologs were identified for the ancient vascular plant species *Selaginella moellendorffii* compared to three homologs in the basal angiosperm *Amborella trichopoda*. The basal monocot *Spirodela polyrhiza*, and basal eudicot *Aquilegia coerulea* had three NRAMP copies each. These copies underwent lineage specific expansion and led to a maximum number of 10 NRAMPs in the monocot species *Panicum virgatum* and 13 NRAMPS in the eudicot species *Glycine max*. As regards cacao, five NRAMP homologs were identified; these comprise Thecc1EG035168, Thecc1EG034751, Thecc1EG000729, Thecc1EG035174 and Thecc1EG027424, hereafter designated as TcNRAMP1, TcNRAMP2, TcNRAMP3, TcNRAMP5 and TcNRAMP6, respectively.

#### Phylogeny

**Figure 1:**
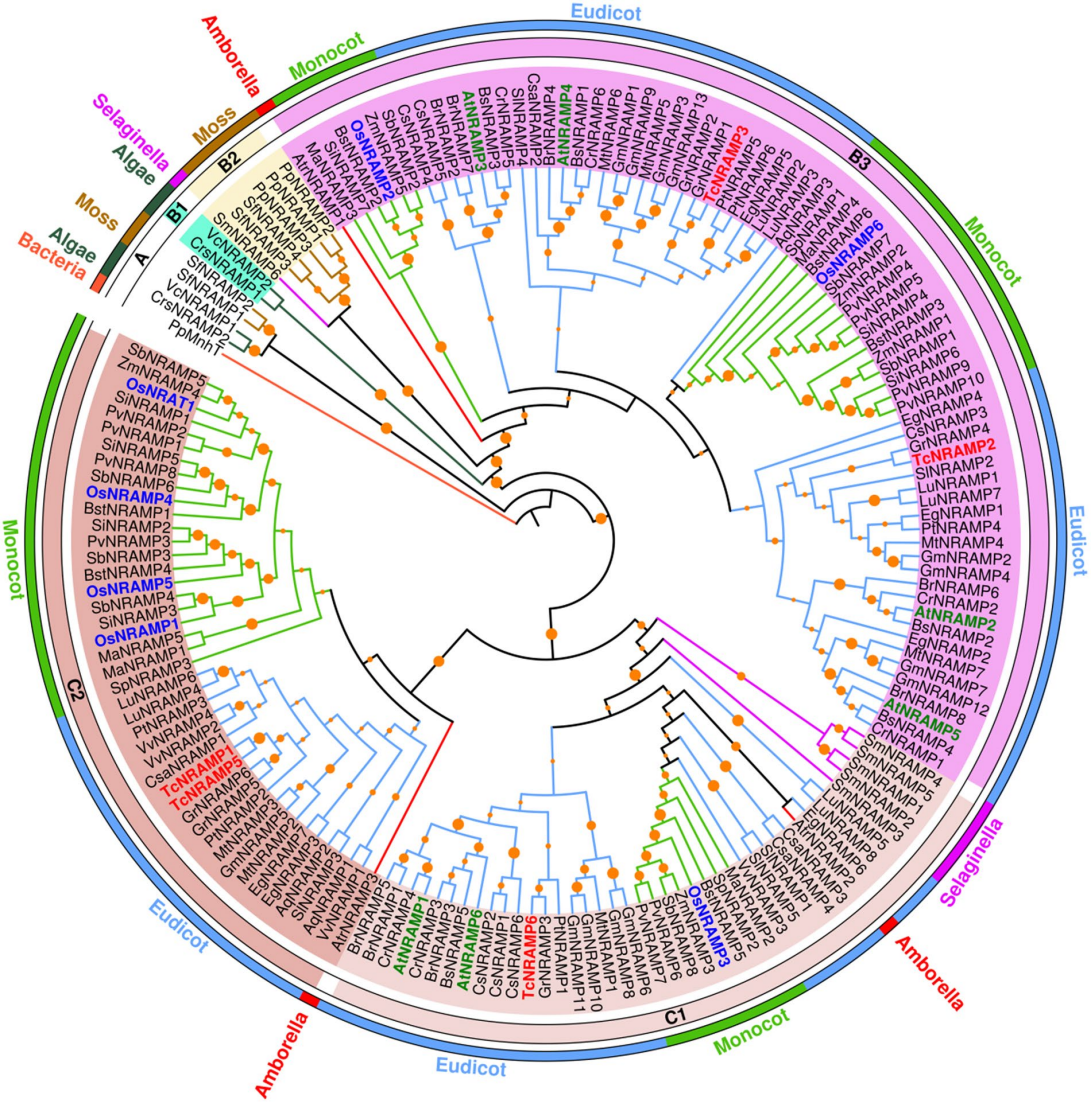
A phylogenetic tree of the NRAMP family in green plants. Maximum Likelihood tree based on the JTT matrix-based model was generated in MEGA7 using the full-length amino acid sequences of the 170 NRAMP proteins from two algae and 28 plant species. The letters (A–C) represent the main clusters. Orange circles on nodes represent bootstrap confidence values, derived from 1000 replications. Names of the species are abbreviated with a two/three-letter code. Number following NRAMP represents multiple members within a single species. *Arabidopsis*, rice and cacao NRAMPs are shown in coloured and bold. Name of the species, abbreviations and corresponding gene loci are given in the Supplemental Table S2.

The NRAMPs phylogenetic tree had strong support for the major nodes (Fig. 1). Based on its topology, the inferred phylogenetic tree revealed three main clusters denoted as A, B and C. Algae and the moss *S*. *fallax* NRAMPs formed the strongly supported basal cluster A, distinct from the derived major clusters B and C. Cluster B had representatives of NRAMP homologs from all selected viridiplantae species, whereas cluster C was formed by NRAMPs exclusively from vascular plants. The strong support for clusters B and C derived from cluster A suggests that all the land plant NRAMP genes have evolved from same ancestor. Cluster B can be further divided into sub-clusters B1, B2 and B3. The second copy of NRAMP homologs from both algae species formed group B1. Five NRAMP homologs from mosses (*P*. *patens* and *S*. *fallax*) and a homolog from spike moss (*S*. *moellendorffii*) were nested in sub-cluster B2. The angiosperm-exclusive B3 sub-cluster, derived from the primitive angiosperm (*A*. *trichopoda*), contained 74 NRAMP homologs, largely from eudicots. The basal monocot and eudicot species (*S*. *polyrhiza* and *A*. *coerulea*) shared one copy of NRAMP whereas *Musa acuminata* and *Solanum lycopersicum*, the adjacent clade in the Phytozome evolutionary tree, contributed two copies in sub-cluster B3, which subsequently expanded with lineage in modern species. TcNRAMP2 and 3, AtNRAMP2 through 5, and OsNRAMP2 and 6 also grouped in sub-cluster B3. Cluster C comprised NRAMP homologs from vascular plants. The cluster was subdivided into sub-clusters C1 and C2 with strong bootstrap support. AtNRAMP1 and 6 formed a predominantly eudicot cluster C1 with 40 homologs from other plant species including OsNRAMP3 and TcNRAMP6. The angiosperm exclusive sub-cluster C2 contained 43 NRAMPs with nearly equal representation from monocots and eudicots. TcNRAMP1 and 5 clustered here with OsNRAMP1, 4 and 5. Both sub-cluster C1 and C2 primarily had one copy each in primitive angiosperm (*A*. *trichopoda*) and basal monocot species (*S*. *polyrhiza*), which then underwent lineage specific expansion.

#### Conserved motif analysis

**Figure 2:**
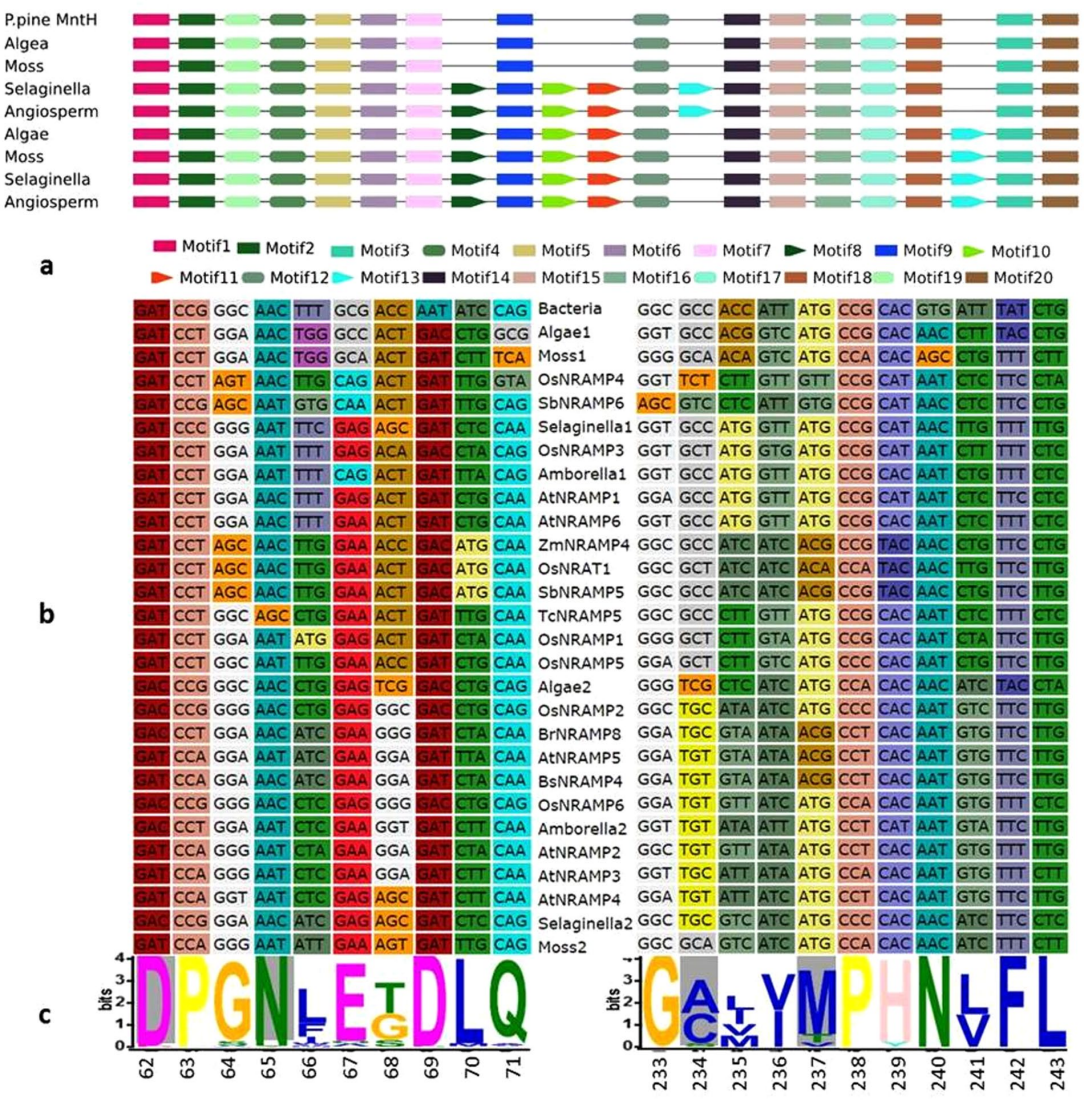
Pattern of conserved motifs of the NRAMP protein and their association with phylogenetic relationship. (**a**) Conserved motifs of NRAMP proteins were identified in 171 sequences from selected species of Bacteria, algae, moss, spike moss (*Selaginella*) and angiosperms using the MEME search tool. Consensus and discriminative motifs are indicated by rectangular box and wedge shapes, respectively. The motif matches are shown with a cut-off p-value less than 0.00001. (**b**) Multiple alignment of coding nucleotide sequences of motif1 and motif9 was determined based on their corresponding amino acid translations using TranslatorX server. (**c**) Sequence logo of the motif1 and motif9 was generated by the WebLogo application. Four conserved residues of the substrate biding site are shaded grey.

Motif analysis by MEME software captured 20 conserved motifs that discriminated 170 NRAMPs, including that from the bacterium MntH, into three distinct groups (Fig. 2a). The NRAMPs from all viridiplantae species in the study formed a distinct group having all 20 conserved motifs. A second group of NRAMPs exclusively from vascular plants differed from the first group by a change in the position of motif13. Bacteria, algae and moss (*P*. *patens*) lacked motif 8, 10, 11 and 13 and formed a third group.

### Structural comparison of NRAMPs from cacao, *Arabidopsis* and rice

#### Primary protein characteristics

The predicted structure and chemical properties of the identified cacao NRAMP proteins were comparable with those from *Arabidopsis* (At) and rice (Os) (see Supplementary Table S1). The cacao NRAMP proteins comprised from 510 to 557 amino acids, with the value of Isoelectric point (IEP) ranging from 4.98 to 8.29. The occurrence of transmembrane domains (TMD) predicted by TMHMM identified all five homologs as transmembrane proteins with 11 to 13 TMD.

#### Conserved motif architecture

**Figure 3:**
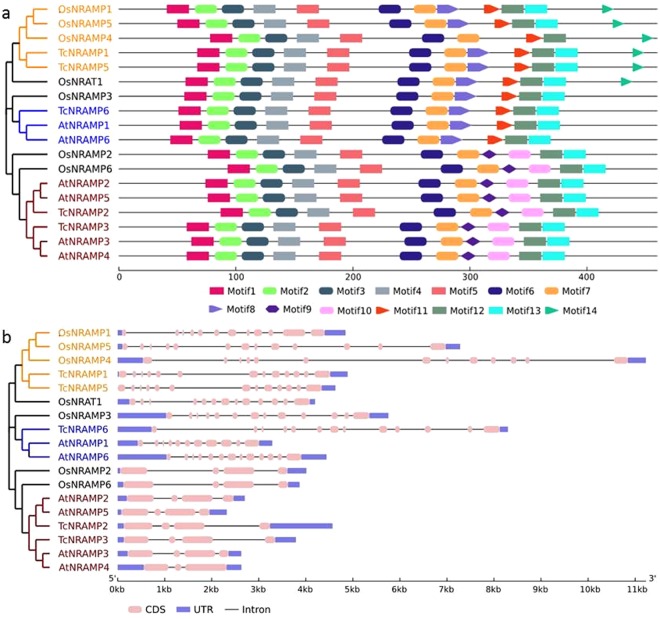
Conserved motif architecture and gene structure of cacao, Arabidopsis and rice NRAMP homologs. (**a**) Pattern of conserved motifs of cacao, *Arabidopsis* and rice NRAMP proteins identified using the MEME search tool. The motif matches are shown with a cut-off p-value less than 0.00001. Shapes represent conserved motifs, whereas black lines indicate non conserved regions. Conserved and non-conserved regions are exhibited proportionally. Scale at bottom is drawn on the *Arabidopsis* NRAMP1 protein. (**b**) Exon/intron organization of cacao, *Arabidopsis* and rice *NRAMP* genes. The blue rectangles represent 5′ and 3′ UTR, the pink round-cornered rectangles indicate exons, and the black lines indicate introns. The sizes of exons and introns can be estimated using the scale at bottom. The Maximum Likelihood tree presented in both figures was generated using the JTT matrix-based model in MEGA7 from full-length amino acid sequences of the 18 cacao (Tc), *Arabidopsis* (At) and rice (Os) NRAMP proteins.

The relationships established in the phylogeny were strongly supported by motif pattern and gene structure analysis. Motif analysis by MEME software identified 14 conserved motifs that discriminated 18 NRAMPs into three distinct groups (Fig. 3a). Motifs 9 and 11 discriminated cluster A and B whereas Motif 9 split cluster A into A1 and A2.

#### Chromosomal location and gene structure

*TcNRAMP1* and *5* are located on chromosome 8, whereas *AtNRAMP1* and *6*, and *OsNRAMP1* and 5 are located on chromosome 1 and 7, respectively. *TcNRAMP2* is also located on chromosome 8, whereas *TcNRAMP2* and *6* are located on chromosome 1 and 6, respectively. Gene structure analysis of the *NRAMP* gene family revealed two distinct groups (Fig. 3b). The group A comprised *OsNRAMP1*, *3*, *4*, *5*; *AtNRAMP1*, *6*; and *TcNRAMP1*, *5*, *6* with the distinct characteristic of having 12 to 13 exons. In contrast, *OsNRAMP2* and *6*; *AtNRAMP2*, *3*, *4* and *5* and *TcNRAMP2* and *3* contained three to four exons. Members of the same cluster varied for intron/exon organization. Exons are more widely dispersed with larger introns in Os*NRAMP5* compared to its highly conserved homolog *OsNRAMP1*. Similarly, *AtNRAMP3* had four exons compared to three exons in the highly similar *AtNRAMP4*.

### Gene family expansion and comparative syntenic analysis

**Figure 4:**
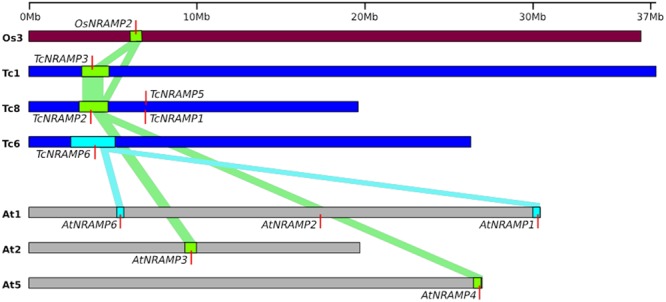
Chromosomal distribution and synteny of *NRAMP* genes. Cacao (Tc), *Arabidopsis* (At) and rice (Os) chromosomes are depicted as blue, grey and brown bars, respectively. Vertical red lines represent *NRAMP* genes. Coloured bars connected syntenic regions between cacao, *Arabidopsis* and rice.

To investigate the effect of duplications on the expansion of NRAMP gene family, we obtained syntenic data of cacao, *Arabidopsis* and rice from PGDD (Plant Genome Duplication Database). Analysis of cacao data revealed one tandem duplicated pair of paralogs *TcNRAMP1* and *5* tightly clustered on chromosome 8, and one segmental duplication pair of cacao *NRAMP* paralogs (*TcNRAMP2-TcNRAMP3*). Two segmental duplication pairs (*AtNRAMP1-AtNRAMP6* and *AtNRAMP3-AtNRAMP4*) were found in *Arabidopsis*. One syntenic block containing the paralogous pair *OsNRAMP2* and 6 was found in rice (Fig. 4). No tandem duplicated pair of *NRAMP* genes was found in *Arabidopsis* and rice. For elucidation of duplication events, we utilized the Ks values for each paralogous pair within a syntenic block. The Ks values for paralogous pairs in cacao (*TcNRAMP2-TcNRAMP3*) and *Arabidopsis* (*AtNRAMP3-AtNRAMP4*) were 1.46 and 1.63, respectively, which implies that these paralog gene pairs may have evolved from the ancient hexaploidization event. Other paralogous gene pairs in *Arabidopsis* (*AtNRAMP1-AtNRAMP6*) and rice (*OsNRAMP2-OsNRAMP6*) had Ks values of 0.88 and 0.89, respectively, which suggests their origin occurred later from species specific lineage segmental duplication events. Comparative analysis of syntenic data from cacao, *Arabidopsis* and rice showed that *TcNRAMP6* corresponds to a pair of recently duplicated *Arabidopsis* paralogs *AtNRAMP1-AtNRAMP6*. The *OsNRAMP2* gene corresponds to *TcNRAMP2* and 3 and *AtNRAMP3* and *4* genes (Fig. 4). In summary, syntenic analysis of the cacao *NRAMP* gene family revealed three distinct types i.e. Type1-*TcNRAMP2*-*TcNRAMP3* (segmental duplicates), Type2-*TcNRAMP6*, and Type3-*TcNRAMP1*-*TcNRAMP5* (tandem duplicates). Examination of the phylogenetic tree topology (Fig. 1), showed that type 1, 2 and 3 are located in the vascular plants exclusive sub-clusters B3, C1 and C2, respectively.

### Comparative expression analysis of cacao, *Arabidopsis* and rice *NRAMPs* in different organs

To investigate functional synteny among the identified *NRAMP* paralogs in the three species, expression profiles of *NRAMP* homologs in cacao were determined experimentally, whereas Genevestigator was used to obtain transcriptome data of *Arabidopsis* and rice *NRAMP* genes.

Four diverse organ types including root, mature leaf, unopened flower bud and bean were subjected to RT-PCR to obtain expression pattern and relative abundance of cacao *NRAMP* transcripts. The reference gene Acyl Carrier Protein (ACP1) was constitutively expressed across the various tissues, thus proving its suitability as a comparator (Fig. S1). Among the target genes, *TcNRAMP1* and *5* were specifically expressed in root, unopened flower bud and bean with a comparatively higher level of expression in root, suggesting their specificity to this organ. The *TcNRAMP6* gene was also predominantly expressed in root and unopened flower bud among the tissues examined and its transcripts were found in low abundance in other tissues studied. The RT-PCR analysis revealed uniform expression of *TcNRAMP2* and *3* across the various organs.

Transcriptome data of *Arabidopsis* and rice *NRAMP* paralogs retrieved from Genevestigator are depicted in Supplementary Fig. S2. Comparison of the expression profile of cacao *NRAMP* paralogs with *Arabidopsis* and rice transcriptome data revealed a distinct pattern. Most of the paralogs from the three species grouped in cluster B3 either showed higher expression in leaf and reproductive tissues compared to root or were expressed universally across the organs. Root-specific *NRAMP* paralogs were grouped in cluster C2 and C3. The *TcNRAMP1* and *5* genes, which previously showed strong structural homology with the rice root specific *NRAMP5*, also followed a similar pattern of expression. Similarly, *AtNRAMP1*, which tightly clustered with *TcNRAMP6* in the phylogenetic tree, was also highly expressed in root compared to leaf and reproductive organs. The two genes *TcNRAMP2* and *AtNRAMP2*, which clustered together, were expressed universally across the organs. Similarly, *TcNRAMP3* showed association with its *Arabidopsis* counterpart *NRAMP4* that demonstrated constitutive expression.

### Expression pattern of cacao *NRAMPs* under metal cation deficiency

**Figure 5:**
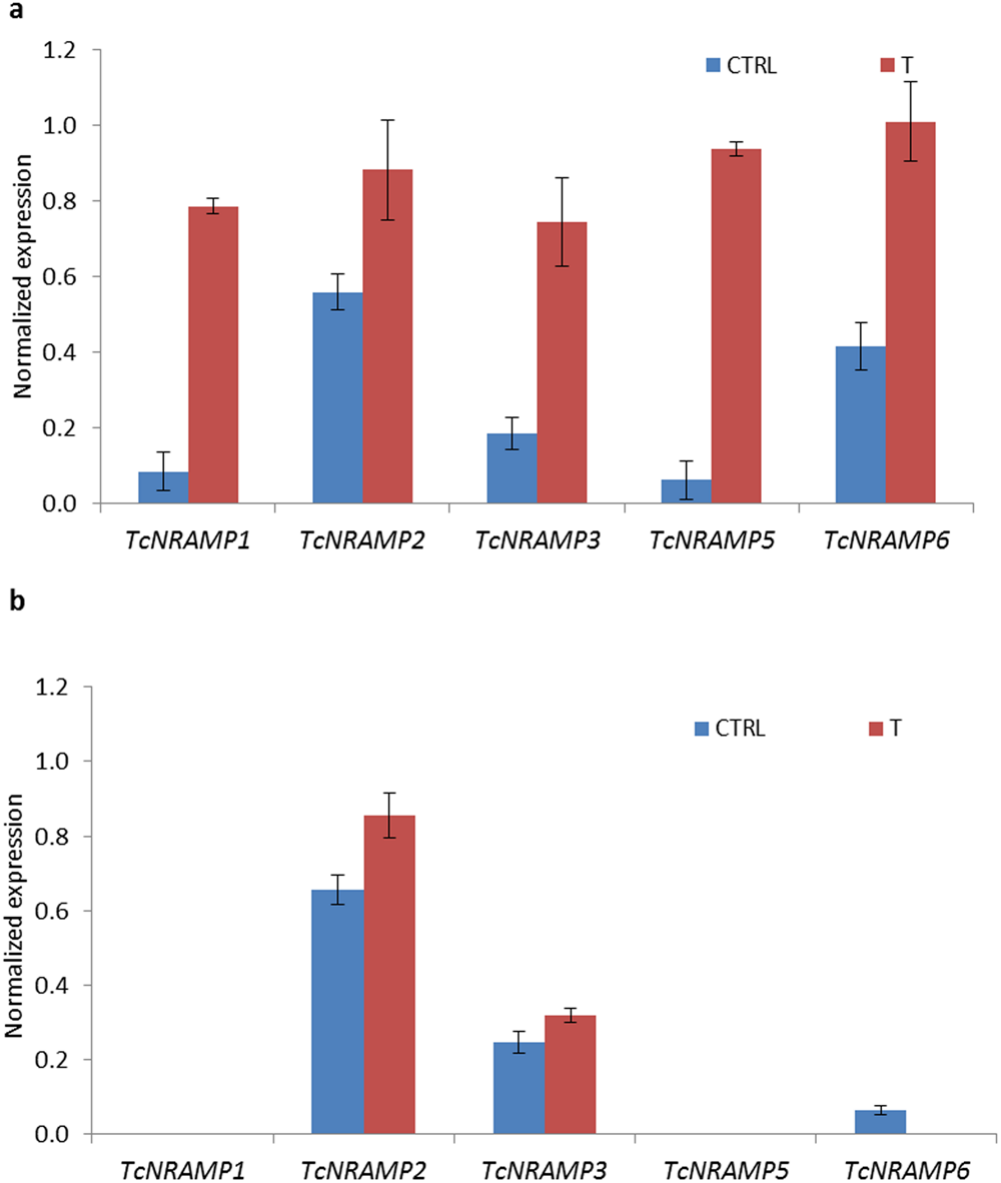
Expression analyses of cacao NRAMP gene family. Normalized expression values of five cacao NRAMPs under Fe^2+^, Zn^2+^, Mn^2+^ containing (CTRL) and deficient (T) conditions in roots (**a**) and shoots (**b**). Pattern of expression was determined in hydroponically grown seedlings using qRT-PCR. Acyl Carrier Protein gene was utilised as the reference gene to normalize the expression values. Data are means ± SD of three biological replicates.

**Figure 6:**
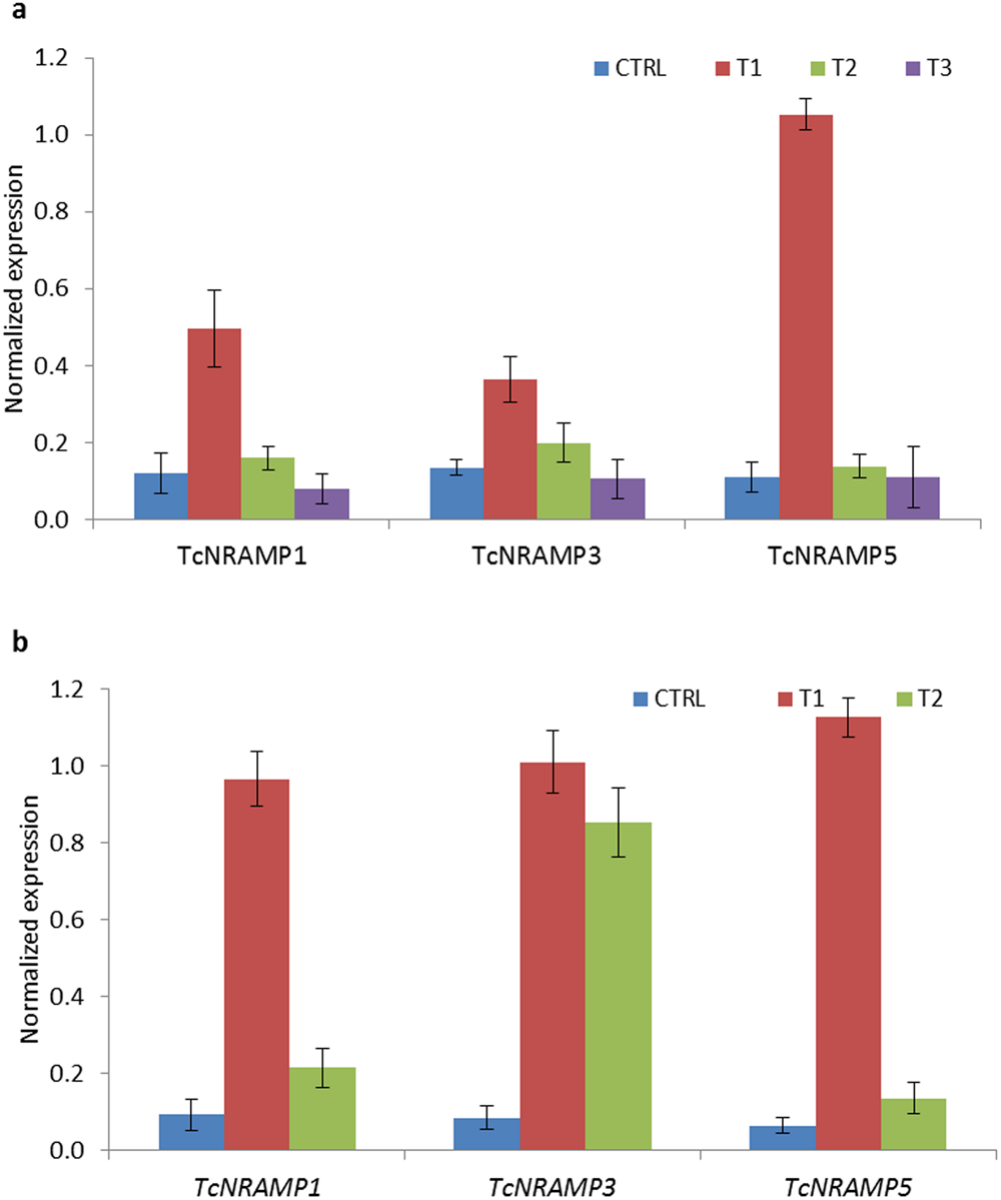
Expression analyses of selected cacao *NRAMP* genes. (**a**) Normalized expression values of *NRAMP1*, *3* and *5* genes in root tissue of cacao seedlings grown under four nutrient conditions i.e. standard 0.5X Hoagland solution (CTRL), CTRL excluding Fe^2+^ (T1), CTRL excluding Mn^2+^ (T2) and CTRL excluding Zn^2+^ (T3). (**b**) Normalized expression values of *NRAMP1*, 3 and *5* genes grown in standard 0.5X Hoagland solution (CTRL), excluding Fe^2+^, Zn^2+^, Mn^2+^ (T1) and excluding Fe^2+^, Zn^2+^, Mn^2+^, but including 20 µM Cd^2+^ (T2). Pattern of expression was determined using qRT-PCR. Acyl Carrier Protein was utilised as the reference gene to normalize the expression values. Data are means ± SD of four biological replicates.

To determine the role of the cacao NRAMP gene family in cation transport, we used qRT-PCR to conduct gene expression studies on seedlings grown hydroponically with various combinations of nutrient cations. In the first experiment, expression of the cacao NRAMP genes was assessed under two different hydroponic growth conditions i.e. seedlings grown in standard Hoagland solution and modified Hoagland solution, which lacked Fe^2+^, Zn^2+^, and Mn^2+^. Overall, *TcNRAMP1*, *5* and *6* transcripts were predominantly expressed in roots, whereas *TcNRAMP2* and *3* were constitutively expressed in leaf and root. These findings are consistent with the RT-PCR results. Root specific cacao NRAMP genes exhibited a high degree of sensitivity to nutrient cation deficiency (Fig. 5a). Expression of *TcNRAMP5*, *1* and *6* increased 15, 10 and 2.5 fold, respectively, in nutrient cation deficient condition compared to control. *TcNRAMP3* also showed significant transcript sensitivity to nutrient deficiency in roots; however, its expression remained stable in leaf. Cation exclusion did not significantly influence expression of *TcNRAMP2* in either organ (Fig. 5a,b). The high degree of sensitivity of cacao *NRAMPs* to nutrient cation deficiency found here, suggests their putative role in cation uptake. However, assessment of the individual effect of each nutrient cation on expression requires further investigation. Therefore, in a subsequent experiment, we attempted to separate the individual effect of each of the divalent cation on the expression of *TcNRAMP1*, *3* and *5*, which were found to be most sensitive to combined Fe^2+^, Zn^2+^ and Mn^2+^ deficiency. The expression data revealed that deficiency of Zn^2+^ and/or Mn^2+^ did not trigger any change in expression of any of the three genes compared to control. However, a significant increase in gene expression was detected under the iron deficient treatment (Fig. 6a). *TcNRAMP5*, *1* and *3* demonstrated a respective 9, 4 and 3 fold increase in expression in Fe^2+^ deficient condition compared with the control. Taking account of the significant change in expression under Fe^2+^ depleted condition, it is suggested that these genes may have a role in Fe^2+^ transport in cacao.

### Expression response of cacao *NRAMPs* to cadmium

The NRAMP gene family is fundamentally involved in uptake and transportation of essential nutrient elements like Fe^2+^ and Mn^2+^. However, the encoded proteins exhibit limited selectivity for divalent metal cations and some NRAMP proteins can also mediate Cd^2+^ transport. Cadmium enters into root cells as an opportunistic hitchhiker on poorly specific transporters. The nutrient cations and Cd^2+^ are taken up through roots and then transported to other plant organs. This fact implies that the genes encoding transporters involved in uptake of Cd^2+^ from soil may be expressed more highly in roots compared to other plant organs. The Fe^2+^ and Mn^2+^ NRAMP transporters can also mediate Cd^2+^ transport. To investigate relative expression between species, we compared the pattern of expression under metal deficient conditions for *Arabidopsis* and rice root specific NRAMPs retrieved from Genevestigator (Fig. S3) with data for the cacao root-specific NRAMPs from the present study. Analyses of microarray expression data revealed high sensitivity of *Arabidopsis* Cd^2+^ transporters *AtNRAMP1* and *4* to Fe^2+^ deficient conditions, with *AtNRAMP4* found to be more sensitive than *AtNRAMP1* to conditions of Fe^2+^ deficiency. The extent of sensitivity also increased with an increase in the duration of iron deficiency. Similarly, in rice, the root-specific iron transporter OsNRAMP1 is also involved in cadmium transport. As described previously, root-specific *TcNRAMP1* and *5* both exhibited a high degree of sensitivity to Fe^2+^ deficiency, suggesting their putative role in Fe^2+^ uptake. In view of the significant structural and functional homology of these cacao NRAMPs with the functionally characterized *Arabidopsis* and rice Cd^2+^ transporters, they may have a similar role in cadmium uptake. To test the hypothesis, we conducted a third study to determine the transcript accumulation of TcNRAMP1, 3 and 5 in response to cadmium stress. For separation of the individual effect of Cd^2+^ from other nutrient cations, expression of *TcNRAMP* genes was assessed under three different hydroponic growth conditions i.e. seedlings grown in standard Hoagland solution (CTRL), CTRL lacking Fe^2+^, Zn^2+^, Mn^2+^ (T1) and T1 supplemented with Cd^2+^ (T2). Root-specific *TcNRAMP* genes exhibited a high degree of upregulation in expression in nutrient cation deficiency (Fig. 6b). Interestingly, the expression level of the genes in a nutrient deficient conditions supplemented with Cd^2+^ (T2) was significantly reduced, to the level of expression detected under control conditions. Expression of *TcNRAMP3* was also induced in response to nutrient cation deficiency; however, addition of Cd^2+^ did not show any effect.

### Functional characterization of cacao NRAMP genes

#### Cloning of *TcNRAMPs*

For functional characterization, the complete opening reading frame (ORF) of the five cacao *NRAMPs* were cloned by the RT-PCR approach. Interestingly, restriction analyses showed the presence of more than one cDNA clone for *TcNRAMP5*. Sequencing of *TcNRAMP5* clones revealed a fully spliced 1671 bp ORF, and a 1527 bp misspliced variant containing partial deletion of exons 10 and 12 and complete deletion of exon 11. However, the deletion did not alter the reading frame, and the clone encoded a 509 amino acid protein with a deletion from aa 411 to aa 458 compared with the full-length protein. The variant is designated as *TcNRAMP5s*. Clones generated for *TcNRAMP1*, *2*, *3* and *6* contained full length coding sequences.

#### Heterologous expression in yeast

**Figure 7:**
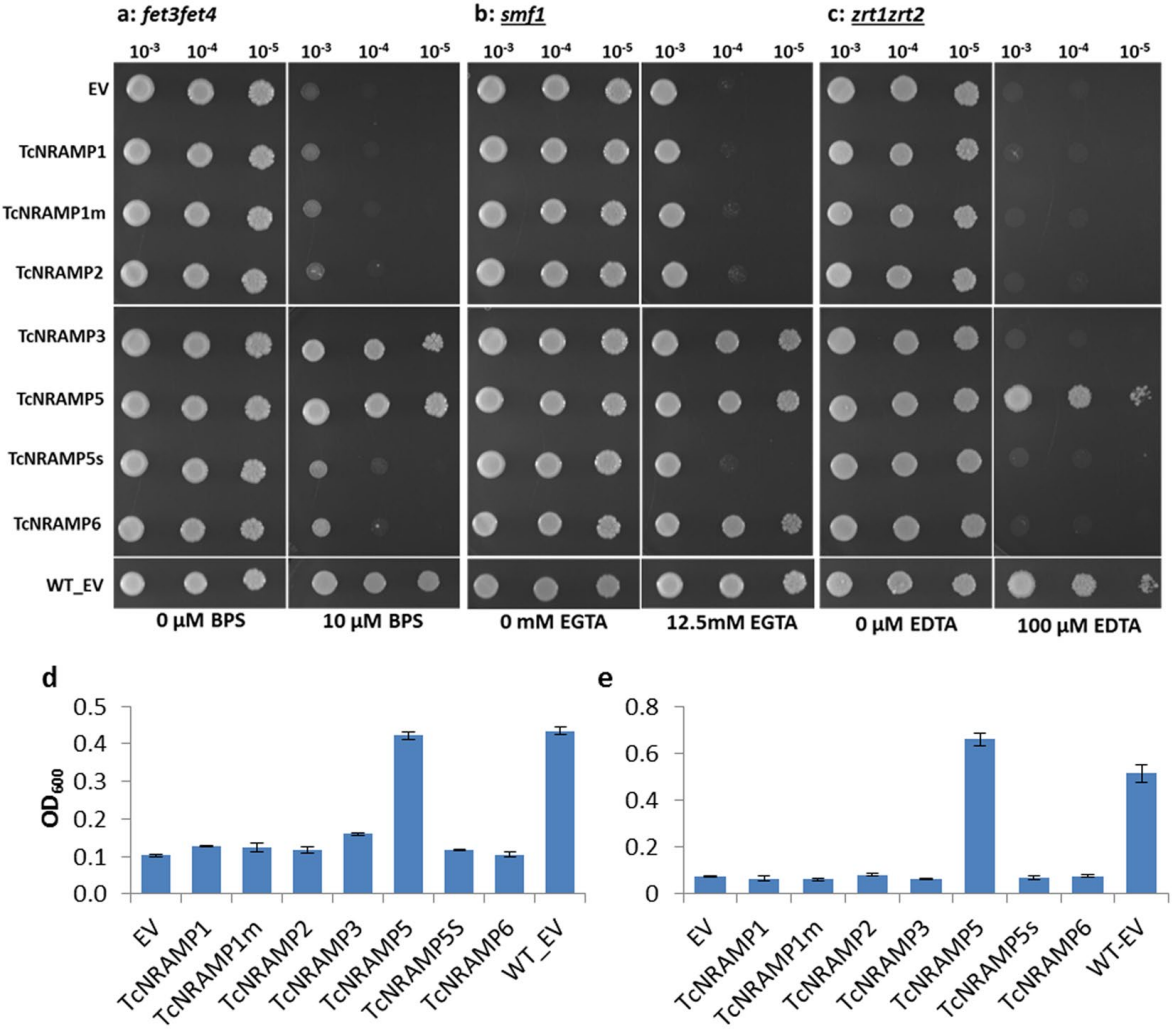
Functional analysis of cacao NRAMP genes in yeast. Functional complementation for uptake of Fe^2+^, Mn^2+^ and Zn^2+^ in the yeast strains DEY1453 (*fet3fet4*), YOL122C (*smf1*) and ZHY3 (*zrt1zrt2*). Different yeast strains expressing *TcNRAMP1*, *1m* (mutated version of *TcNRAMP1*), *2*, *3*, *5*, *5s* (splice variant of *TcNRAMP5*), *6* or empty vector pDR195 (EV) were cultured on synthetic defined (SD)-Ura plates containing 2% glucose in presence or absence of respective metal chelator. Transformed *fet3fet4* cells were spotted on the plate (pH 4.0) supplemented with 10 μM FeCl_3_ or 10 μM Fe chelator (**a**) and also grown (**d**) in low iron liquid medium (LIM10). Transformed *smf1* cells were grown on the plate (pH 5.2) supplemented with 100 μM MnSO_4_ or with 12.5 mM Mn chelator EGTA (**b**). The yeast mutant strain ZHY3 (*zrt1zrt2*) expressing the genes were assessed on the plate (pH 5.8) supplemented with 100 μM ZnCl_2_ or with 100 μM of metal chelator EDTA, 10 μM each of FeCl_3_ and ZnCl_2_ (**c**) and also grown (**e**) in low zinc liquid medium (LZM100). The wild type yeast strain DY1457 transformed with the empty vector pDR195 (WT-EV) was included as a positive control. Data are means ± SD of three biological replicates.

The role of the cacao NRAMPs in Fe^2+^, Zn^2+^ and Mn^2+^ transport was determined by testing for the complementation of the phenotype of the double mutant *S*. *cerevisiae* strain DEY1453 (*fet3fet4*), ZHY3 (*zrt1zrt2*) and single mutant *smf1*, respectively. The strain DEY1453 is defective for both high- and low-affinity Fe^2+^ uptake systems^6^. Similarly, the ZHY3 strain lacks a functional copy of the high- and low-affinity Zn^2+^ transporters, ZRT1 and ZRT2, respectively^7^. The Mn^2+^ mutant strain *smf1* lacks the *SMF1* gene, essential for high affinity Mn^2+^ uptake. Consequently, these mutants need a much higher amount of the respective metal for their growth compared to the parental wild-type strain. For the Fe^2+^ assay, the transformed mutant strain *fet3fet4* and wild type cells were grown on SD medium either supplemented with the Fe^2+^ chelating agent bathophenanthroline disulfonate (BPS) or without BPS (control). The control plate showed good growth for all yeast cells. In Fe^2+^ limited conditions, out of the five *TcNRAMP*s studied, only cells expressing *TcNRAMP3* and *5* could rescue the phenotype (Fig. 7a). To test the various *TcNRAMP*s for their Mn^2+^ transport activity, the treatment plate was prepared by adding the divalent cation chelator Ethylene glycol-bis(2-aminoethylether)-N,N,N′,N′-tetraacetic acid (EGTA) to the solid SD medium to limit the availability of Mn^2+^. Expression of *TcNRAMP3*, *5* and *6* successfully complemented the phenotype of the *smf1* strain on the treatment plate. The control plate showed uniformly good growth for all yeast cells (Fig. 7b). In the assay of Zn^2+^ uptake, the strain carrying *TcNRAMP5* showed significant Zn^2+^ transport activity (Fig. 7c). *TcNRAMP1*, *2* and splice variant *TcNRAMP5s* failed to show transport activity for any of the metals tested. As expected, wild type strain DY1457 containing the empty vector pDR195 grew well in both conditions, whereas mutant strains transformed with the empty vector pDR195 showed good growth on control plate only. The growth assay conducted on low iron or zinc liquid medium produced the similar results (Fig. 7d,e).

**Figure 8:**
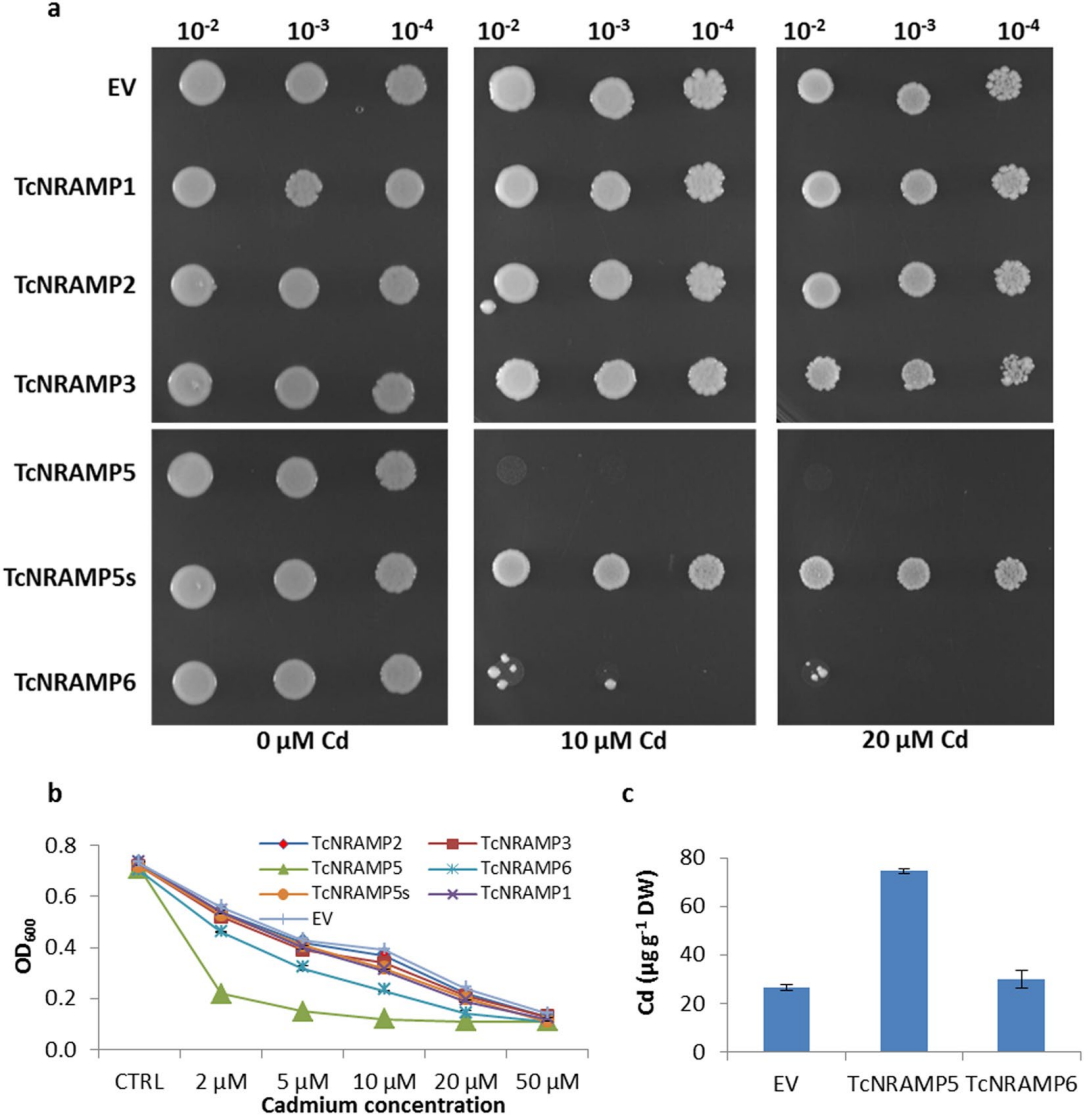
Analysis of cadmium sensitivity conferred by expression of cacao NRAMP genes in yeast. Yeast strain DY1457 carrying *TcNRAMP1*, *2*, *3*, *5*, *5s* (splice variant of *TcNRAMP5*), *6* or empty vector pDR195 (EV) were cultured on synthetic defined (SD)-Ura plates and liquid medium (**b**) in absence or presence of different concentration of CdCl_2_. The plates were incubated at 30 °C for 3 days (**a**). For growth assay in liquid medium (**b**) the cells were grown at 0.001 OD_600_ for 72 h at different concentrations of CdCl_2_ as indicated in the figure. (**c**) Yeast strain DY1457 expressing *TcNRAMP5*, *6* or empty vector pDR195 (EV) were grown in SD-Ura liquid medium supplemented with 2 μM CdCl_2_ for 72 h. The Cd^2+^ accumulation in the cells was determined by inductively coupled plasma mass spectrometry (iCAP™ Q ICP-MS). Data are means ± SD of three biological replicates.

In addition to uptake and transportation of essential nutrient elements, the NRAMP gene family is involved in transport of Cd^2+^ due to poor substrate specificity. Therefore, we investigated cadmium transport ability of TcNRAMPs in yeast at different Cd^2+^ concentrations. For the cadmium uptake test, wild type yeast strain DY1457 was transformed with individual cacao *NRAMP*s including splice variant *TcNRAMP5s*, and empty vector pDR195 and spotted on control and Cd^2+^ containing SD medium agar plates. The yeast cells transformed with *TcNRAMP5* showed absolutely no growth in the presence of 10 µM Cd^2+^, indicating high-affinity Cd^2+^ transport by this protein. However, the splice variant *TcNRAMP5s* failed to show sensitivity to the Cd^2+^. Significant differences in growth of *TcNRAMP6* transformed cells was observed on 10 and 20 µM Cd^2+^ concentrations compared to control (Fig. 8a). Growth of the cells expressing either *TcNRAMP1*, *2* or *3* was not significantly affected by the presence of Cd^2+^, and was quite comparable with growth of cells containing empty vector. For quantitative assessment of growth response to Cd^2+^, we evaluated the transformed yeast cells in liquid SD medium containing five different Cd^2+^ concentrations. Expression of *TcNRAMP5* leads to highly significant sensitivity to Cd^2+^. Growth inhibition of 70% compared to control was recorded for cells expressing *TcNRAMP5* even at the very low Cd^2+^ concentration of 2 µM. Higher Cd^2+^ concentrations of 5 µM and above completely diminished growth of the cells (Fig. 8b). The yeast cells transformed with *TcNRAMP6* also showed mild inhibition of 34% in relative growth at 2 µM Cd^2+^ concentration, which gradually increased to 45, 67, 80 and 84% at 5, 10, 20 and 50 µM Cd^2+^ concentrations, respectively. The growth inhibition of cells expressing either *TcNRAMP1*, *2* or *3* or splice variant *TcNRAMP5s* was comparable with the cells containing empty vector. In addition to the sensitivity test, accumulation of Cd^2+^ was quantified in the wild type yeast strains expressing *TcNRAMP5* or *6*; these showed sensitivity to Cd^2+^ that was similar to that of control cells transformed with the empty vector. As the strain expressing *TcNRAMP5* revealed very high sensitivity to the Cd^2+^, the cells were exposed to low concentration of 2 µM Cd^2+^ for 72 h. The yeast cells expressing *TcNRAMP5* accumulated three times more Cd^2+^ than those with only the vector. Despite showing hypersensitivity to Cd^2+^, *TcNRAMP6* expressing cells showed non-significant differences for accumulation of Cd^2+^compared to the control (Fig. 8c). These findings strongly suggest that, TcNRAMP5 is able to transport Cd^2+^ in addition to essential nutrient metal cations.

## Discussion

Natural resistance-associated macrophage proteins (NRAMPs) are reportedly involved in binding and transport of essential metal cations, and exist in all kingdoms of life. Cacao is an economically important crop renowned for its integral role in chocolate and beverage industry. The members of NRAMP family have been identified and functionally characterized in number of plant species including *Arabidopsis*, rice, and soybean^8–12^, however to date such information is lacking in cacao. Here, we searched and aligned *Arabidopsis* NRAMP homologs in selected viridiplantae species to obtain an insight about the evolutionary relationship of NRAMPs among the plant species that included cacao. Then *Arabidopsis* and rice NRAMP transporters, and cacao NRAMPs identified following phylogenetic analysis were subjected to detailed structural predictions and functional analyses.

The genomes of recently evolved plant species genomes contain variable number of NRAMP proteins. Though the basal angiosperm *A*. *trichopoda* had three copies, these copies underwent lineage specific expansion to 10 and 13 copies found here in monocot species *Panicum virgatum* and eudicot species *Glycine max*, respectively. An informatics search of the cacao genome identified five NRAMP homologs compared to six and seven homologs in *Arabidopsis* and rice, respectively. Gene duplication contributes to expansion and functional diversification of gene families, and may result from tandem duplication, arise through unequal crossing over, or be caused by segmental duplication, including whole genome duplication (WGD) and duplications of large chromosomal regions^13^. Analyses of syntenic data of cacao revealed one tandem duplicated pair of paralogs (*NRAMP1* and *5*) and one segmental duplication (*NRAMP2* and *3*), which led to an increase in copy number to five. However, the three types of cacao NRAMPs (1–5; 2–3; 6) represent three members in the basal angiosperm (*A*. *trichopoda*). All the sequenced angiosperm genomes have undergone ancient and more recent WGD events^14^. *Arabidopsis* has experienced at least three rounds of such events since its divergence from other Brassicales^15^. The cacao genome has not undergone any WGD event since the pan-eudicot triplication^16^. Therefore, the segmental duplication (*TcNRAMP2-TcNRAMP3*) might have arisen from duplications of large chromosomal regions during evolution.

Phylogenetic analysis grouped NRAMP proteins from selected viridiplantae species into three clusters. Algae and moss NRAMPs formed a distinct cluster. Cluster B had representatives of NRAMP homologs from all selected viridiplantae species, whereas cluster C was formed by NRAMPs exclusively from vascular plants. Three NRAMP copies in the basal angiosperm *A*. *trichopoda* and the basal monocot *S*. *polyrhiza* nested in three distinct clusters, which suggests that each cluster had a common ancestor (Fig. 1). The five NRAMP proteins identified in cacao clustered into two groups B and C with cluster C being further branched into two distinct sub-clusters. The phylogenetic analysis of NRAMP proteins in *Arabidopsis*^17^, rice^10^ and soybean^12^ have revealed the same clustering pattern found here in cacao. Since gene structures are reported to be conserved among paralogs and homologs in many gene families, these data may provide insights into the evolutionary history of NRAMP gene family.

The comparative analysis of conserved motifs and intron/exon organization in cacao, *Arabidopsis*, and rice (Fig. 3a,b) corresponded to the phylogenetic groups. The number of exons/introns was highly conserved within each of the two subfamilies. For instance, *TcNRAMP3* and *4*; *AtNRAMP2* through *5;* and *OsNRAMP2* and *6* in cluster B contain 3–4 exons, whereas the other subfamily represented by *TcNRAMP1*, *5* and *6*; *OsNRAMP2* through *5*; and *AtNRAMP1* and *6* have 12 to13 exons.

Determination of tissue specificity of gene expression may aid the prediction of physiological function of the respective gene. Expression profiling of cacao *NRAMP* genes revealed a diversified pattern of expression in different organs. *TcNRAMP1*, *5* and *6* were primarily expressed in roots whereas *TcNRAMP2* and *3* were uniformly expressed across the organs (Fig. S; Fig. 6a,b). The root specific expression of *TcNRAMP1*, *5* and 6 implies their role in uptake of metals from the external solution. We also retrieved microarray expression data for *Arabidopsis* and rice from Genevestigator (Fig. S2) to compare it with cacao. The expression pattern also supported the relationships found among the three species in the phylogenetic and gene structure analysis. For example, most of the paralogs grouped in cluster B either showed higher expression in leaf and reproductive tissues compared to roots or were expressed universally across the oragns, whereas paralogs in cluster C expressed exclusively in roots. The relationship found here in microarray data is also supported by tissue specific expression determined through qRT-PCR in *Arabidopsis*^8^ and rice^10^ NRAMP transporters.

Functional divergence is a primary outcome of gene duplication, and may occur by either neo-functionalization (duplicated gene gains entirely new function compared to the function of ancestral gene) or sub-functionalization, (duplicate gene complements the function of ancestral gene). Having occurred recently, tandem duplicates are more likely to show higher level of complementarity than segmental duplicates^18^. Results of comparative expression analysis of duplicated paralogous pairs predicted in synteny analysis were found to be consistent with sub-functionalization. The cacao tandem duplicated pair (*NRAMP1-NRAMP5*) were co-expressed in root and flower bud whereas segmental duplicate pair (*NRAMP2-NRAMP3*) showed uniform expression.

Expression of NRAMP transporter genes was generally shown to be up-regulated under divalent metal starvation. We also found significant transcript sensitivity to cation deficiency for cacao *NRAMP* genes in roots. The root specific *TcNRAMP1* and *5* transcripts were significantly upregulated under iron deficiency; however, limitation of Zn^2+^ and/or Mn^2+^ did not trigger any change in expression (Fig. 6a). Similar features have been reported for their paralogs in *Arabidopsis* and rice. *AtNRAMP1* was primarily expressed in roots and demonstrated up-regulated expression under Fe^2+^ starvation^8,19^. However, its expression was also upregulated by Mn^2+^ deficiency^20^. The rice paralog *OsNRAMP5* was also predominantly expressed in roots and exhibited significantly high expression under Fe^2+^ limited conditions, but was unaffected by Mn^2+^ deficiency^21^. The protein sequence comparison showed that TcNRAMP1 and 5 had high identity of 72% with OsNRAMP5 compared to 58% identity with AtNRAMP1. These findings suggest that TcNRAMP1 and 5 may have more functional homology with OsNRAMP5 than with AtNRAMP1 in relation to metal transport. *TcNRAMP3* also exhibited highly significant expression in Fe^2+^ limited conditions (Fig. 6a), which corresponded to induced expression of the *Arabidopsis* ortholog *AtNRAMP4* under Fe^2+^ deficiency^8^. *AtNRAMP6* is expressed mainly in shoot and dry seeds and acts as an intracellular metal transporter^11^. *TcNRAMP6*, which showed mild sensitivity to exclusion of all three metal cations, failed to show sensitivity specific to deficiency of any particular metal and might have a role in intracellular metal transporter. A recent study has reported detailed characterization of *AtNRAMP2*. The gene expressed constitutively in root and shoot but the expression was not altered by exclusion of Fe^2+^, Zn^2+^or Mn^2+^ in either organ^22^. Similarly, we also did not find any significant influence of cation exclusion on expression of the *NRAMP2* ortholog in cacao (Fig. 6a).

Cadmium has long been recognized as a major health concern to humans. Plants have a tendency to uptake Cd^2+^ from soil and accumulate it in edible parts, which represent the main source of Cd^2+^ in human food^23^. Cadmium, being toxic, is not vital for plant growth, and therefore at first sight, there is expected to be no selection pressure to favour a Cd^2+^ specific transporter. The question remains, however, as to whether Cd^2+^ accumulation might have an indirect benefit to plant performance, for example by inhibiting pests and/or pathogens^24–26^, and therefore there might indeed be selection in favour of Cd^2+^ uptake. The evidence in support of such a theory comes from the increasing number of studies^27–32^ that have identified the inhibitory effect of leaf cadmium on feeding and other behaviour of herbivorous caterpillars such as *Spodoptera litura* and *Lymantria dispar*.

The NRAMPs have broad substrate specificity and have been reported to transport cadmium in addition to Mn^2+^, Fe^2+^ and Zn^2+^ in *Arabidopsis*, rice and many other plants^8,10,21,33–36^. Significant structural and transcriptional similarity of TcNRAMP1, 3 and 5 with the functionally characterized *Arabidopsis* and rice Cd^2+^ transporters implies their possible role in Cd^2+^ uptake. To test the hypothesis, we conducted an expression study to determine the transcript accumulation of *TcNRAMP1*, *3* and *5* in response to Cd^2+^ stress. The expression studies on response of *NRAMPs* to cadmium usually compare conditions where plants are grown in medium containing nutrient cations in the presence and absence of cadmium^10,35^. As we know that Cd^2+^ and nutrient cations have common transporters, a significant impact of Cd^2+^ on expression of these transporters cannot be expected in the presence of nutrient cations. For separation of the individual effect of Cd^2+^ from nutrient cations, expression of *TcNRAMPs* found to be sensitive to metal deficiency was assessed in the presence and absence of nutrient cations, and absence of nutrient cations in media containing Cd^2+^. All three genes, as expected, showed high sensitivity to nutrient cation deficiency; however, addition of Cd^2+^ drastically reduced the expression of *TcNRAMP1* and *5* to the level detected under control conditions (Fig. 6b). Differential expression of these genes in plants grown both with and without Cd^2+^ suggests their role in cadmium transport. As previously discussed, Cd^2+^ enters the root cell opportunistically through poorly specific transporters. It can be argued that the control system (at present unknown) for *TcNRAMP1* and *5* probably recognized Cd^2+^ as a divalent nutrient cation, which resulted in complete reversal of their extremely high expression sensitivity under Cd^2+^ supplemented conditions. These results are also supported by reported role of closely similar *AtNRAMP1*^8^ and *OsNRAMP5*^10^ in Cd^2+^ transport. Addition of Cd^2+^ did not trigger change in expression of *TcNRAMP3*, which suggests a role in the transport of a metal other than Cd^2+^.

Expression pattern of a gene may not necessarily be informative with respect to its function. Therefore, we cloned five *TcNRAMP*s and expressed them in yeast strains for functional characterization. Results showed that transporters encoded by *TcNRAMP3* and *5* have broad substrate specificity including Fe^2+^ and Mn^2+^. TcNRAMP6 is specific for Mn^2+^ transport (Fig. 7). In addition to nutrient cations, yeast expressing *NRAMP5* and *6* exhibited high sensitivity to Cd^2+^ (Fig. 8a,b). The yeast cells expressing *TcNRAMP5* accumulated three times more Cd^2+^ than the vector only. In contrast, cells expressing *TcNRAMP6* showed the same level of Cd^2+^ accumulation as the control (Fig. 8c). *TcNRAMP1* and *2* failed to show transport activity for the metals tested. The metal transport activity reported previously for *Arabidopsis* and rice NRAMPs supports the present findings for the respective orthologs in cacao. Like TcNRAMP5, OsNRAMP5 and AtNRAMP1 have been implicated in Fe^2+^, Mn^2+^ and Cd^2+^ transport^8,10^. Similarly, heterologous expression of *AtNRAMP6* in yeast enhanced sensitivity to Cd^2+^ without affecting cadmium content in the cells^11^. However, *TcNRAMP3*, an ortholog of *AtNRAMP3* and *4*, showed transport activity for Fe^2+^ and Mn^2+^ but not for Cd^2+^. Recently, it has been reported that the AtNRAMP2 protein is involved in remobilization of Mn^2+^ in Golgi for root growth instead of uptake through roots^22^. Based on structural and expression similarities, it is suggested that TcNRAMP2 may be involved in remobilization rather than uptake of metal cation(s).

Structure–function relationships in the context of substrate preference in the NRAMP family have been demonstrated in recent studies^2,37^. The crystal structure of the bacterium *Staphylococcus capitis* NRAMP transporter (ScaNRAMP) revealed a substrate-binding site that coordinates divalent transition-metal ions including Mn^2+^, Fe^2+^ and Cd^2+^. Four conserved residues including aspartic acid (D)49, asparagine (N)52, alanine (A)223, and methionine (M)226 directly bind the metal substrate. Functional investigations have established that mutation of ion-coordinating residue N52 very strongly reduces its binding affinity for Mn^2+^, Fe^2+^ and Cd^2+^. Inspection of the substrate-binding site in the sequences used in this study revealed that the metal substrate binding residue N52 located in motif1 was conserved in all sequences apart from TcNRAMP1 where serine replaced asparagine (Fig. 2b). TcNRAMP1 and TcNRAMP5 shared 92% similarity in protein sequence and showed similar expression pattern in expression studies; however, TcNRAMP1 failed to show transport activity for the metals tested (Figs 7 and 8), a finding that may be attributed to the mutation at the conserved residue N52 (based on ScaNRAMP numbering) resulting in complete loss of function. In order to test this hypothesis we developed a synthetic version (TcNRAMP1m) of TcNRAMP1 (S52N). Like TcNRAMP1, functional characterization of the TcNRAMP1m in yeast expression system did not show any transport activity for Fe^2+^, Mn^2+^ or Zn^2+^, (Fig. 7) which implies that the N52 residue in cacao NRAMP1 has no role in binding of the metals tested.

Complete/partial loss of function of the NRAMPs have been associated with mutation of highly conserved residues involved in metal selectivity^1,38^ and the truncations leading to major structural changes^35^. The key metal selectivity role of conserved methionine (M226) is well established in bacteria. A methionine-to-alanine substitution reduced binding affinity and transport of Cd^2+^ without affecting binding behaviour of Mn^2+^. However, it enables transport of calcium and magnesium, a finding that suggests that the conserved methionine is essential for transport of low-abundance transition metals in the presence of high-abundance divalent metals such calcium and magnesium^1^. Inspection of the conserved methionine in the sequences we identified here revealed 86% conservation. Among the various protein sequences, OsNRAT1 (NRAMP aluminium transporter 1) and AtNRAMP5 have a methionine-to-alanine substitution, whereas valine has replaced the methionine in OsNRAMP4. Rice OsNRAT1 transports the highly abundant trivalent aluminium metal^39^, hence the substitution of methionine may have led to the diverged function. However, AtNRAMP5 and OsNRAMP4 have not yet been functionally characterized. Phenylalanine (F398 ScaNRAMP numbering) is also highly conserved in NRAMPs and induced mutation of the residue in AtNRAMP4 has reduced its ability to transport Cd^2+^ in yeast^38^. On other hand, a mutant identified in tobacco encoding a truncated NRAMP5 protein showed no transport activity for Mn^2+^ and a weak transport activity for Cd^2+^ compared to wild type^35^. Also, OsNRAMP5 mutants developed using either ion-beam irradiation^40^ or the CRISPR/Cas9 gene-editing system^5^ generated C terminal truncations that showed low uptake of Cd^2+^ without compromising yield. We identified a splice variant among the *TcNRAMP5* clones with partial deletion of exons 10 and 12 and complete deletion of exon11. The variant, which encodes a 510 aa peptide, contains the conserved residues that have been implicated as the substrate binding site; however, it lacks transmembrane domain 10 compared to full length TcNRAMP5 (Fig. S4). In contrast to TcNRAMP5, the splice variant did not show evidence for any metal transport activity when expressed in yeast (Figs 7 and 8); this suggests that a deletion in the C terminal region may cause a complete loss of function despite having the conserved substrate binding site. This result is consistent with previous findings in tobacco^35^ and rice^5^. This finding is also supported by an investigation in yeast that affirms the importance of the entire C-terminal domain in stability and trafficking of membrane protein Pma1 H^+^-ATPase^41^. Taken together, these results imply a role of *TcNRAMP5* in Cd^2+^ uptake in cacao. Identification or induction of loss of function mutations in the gene may help in the development of cacao clones with reduced Cd^2+^.

## Methods

### NRAMP homologs prediction, phylogeny, and gene/protein bioinformatic analyses

A BlastP search was conducted using the *Arabidopsis* metal transporters NRAMP1 to 6 protein sequences as queries to identify orthologs in two algae and 28 other plant species (http://www.phytozome.net/). A List of the sequences is provided in Table S2 in Supplementary Information. Each selected sequence was inspected for the presence of the NRAMP domain (pfam01566) using Pfam (https://pfam.xfam.org/) software. The selected NRAMP sequences were subjected to multiple alignment using MUSCLE^42^. The initial tree was generated by using the Maximum Likelihood method based on the JTT matrix-based model in MEGA7^43^. Bootstrap support was determined from 1000 replicates. The phylogenetic tree was visualized and drawn by iTol^44^.

The isoelectric point and molecular mass of the NRAMP sequences were predicted by the ProtParam tool (http://web.expasy.org/protparam/). Transmembrane domains (TMDs) were predicted by the TMHMM Server v. 2.0 (http://www.cbs.dtu.dk/services/TMHMM/). Genomic sequences of cacao NRAMP genes were downloaded from NCBI and gene structures were constructed on the Gene Structure Display Server (http://gsds.cbi.pku.edu.cn/). The expression data of *Arabidopsis* and rice NRAMPs were downloaded from Genevestigator (https://genevestigator.com/gv/) and heat maps were generated using heatmapper software (http://www1.heatmapper.ca/expression/).

### Plant growth

Cacao clone NA702 was chosen for expression analysis and functional characterization of cacao *NRAMP* genes. For organ specific expression, root, mature leaf, unopened flower bud and bean were obtained from the International Cocoa Quarantine Centre, Reading, UK (http://www.icgd.reading.ac.uk/icqc/). For metal sensitivity expression studies, beans were germinated in compost. Two weeks old seedlings were then transferred to half strength Hoagland solution with the pH adjusted to 5.2. The nutrient solution was aerated for 15 min after every two hours, and renewed every week. Plants were cultured under controlled environment conditions (28/22 °C day/night temperature, 16 h photoperiod with 60% relative humidity). The seedlings were grown in the solution for 21 days and then subjected to different nutrient combinations to determine their effect on the gene expression. In the first experiment, the seedlings were subjected to nutrient deficiency by exclusion of Fe^2+^, Zn^2+^ and Mn^2+^ from the half strength Hoagland solution. In a subsequent experiment conducted to separate the individual effect of each of the divalent cations on the expression of cacao *NRAMPs*, the seedlings were exposed to three nutrient deficient conditions i.e. half strength Hoagland solution excluding: Fe^2+^ (T1), Mn^2+^ (T2) and Zn^2+^ (T3). In the third experiment to determine the transcript accumulation in response to cadmium stress, seedlings were grown in half strength Hoagland solution excluding Fe^2+^, Zn^2+^, Mn^2+^ (T1) and T1 supplemented with 20 µM cadmium chloride (T2). Seedlings grown in half strength Hoagland solution were sampled as the control in each experiment. Each treatment included four biological replicates. Leaf and root tissues ware sampled seven days after application of the treatments. The organs sampled for expression studies were stored at −80 °C prior to subsequent RNA extraction.

### Expression analyses

Total RNA was isolated from various organs using a modified CTAB method, and subsequently purified by the RNeasy® Plant Mini Kit (Qiagen) according the manufacturer’s instructions, and quantified through NanoDrop 2000 Spectrophotometer. Additionally, aliquots of extracted RNA were run on 1.5% agarose gel for quality determination. Isolated total RNA (1.0 µg) was converted into cDNA using High-Capacity RNA-to-cDNA™ kit (Thermo Fisher Scientific). Nucleotide sequences for the reference gene and genes of interest were retrieved from the NCBI GenBank database (http://www.ncbi.nlm.nih.gov) and primers were designed using Primer Blast tool (http://www.ncbi.nlm.nih.gov/tools/primer-blast). The list of primers used in the expression study is given in Supplementary Table S3. RT-PCR was performed in a Veriti Thermal Cycler, whereas the StepOnePlus™ Real-Time PCR system was used for real time RT-PCR. Regarding PCR mix, BioMix^TM^ (Bioline) and PowerUp™ SYBR® Green master mix (Applied Biosystems) were used in RT-PCR and qRT-PCR, respectively. RT-PCR products were run on 2.5% agarose gel stained with ethidium bromide. A relative standard curve assay was used to determine the relative expression of genes of interest in real time analysis. The cacao Acyl Carrier Protein (ACP1, GenBank: TCM025966) was utilised as the reference gene for normalization.

### Cloning of cacao *NRAMPs*

Total RNA isolated from roots was converted into cDNA using SuperScript™ III First-Strand Synthesis SuperMix (Thermo Fisher Scientific) to obtain full length products. Full coding sequence of cacao *NRAMP* genes, including stop codon, was amplified by Phusion Hot Start II High-Fidelity DNA Polymerase (Thermo Fisher Scientific) with the primers (see Supplementary Table S4) containing attB overhang. The *TcNRAMP1* and *5* were cloned into pCR™4Blunt-TOPO® cloning vector and then cloned into pDONR221 donor vector, whereas *TcNRAMP2*, *3* and *6* were directly cloned into the donor vector. The entry clones were produced using Gateway® BP Clonase™ II Enzyme Mix (Thermo Fisher Scientific). Yeast expression vector pDR195 was converted to Gateway destination vector by ligating Gateway® Reading Frame Cassette B at the XhoI cloning site, and designated as pDR195GTW. Expression clones were generated using Gateway® LR Clonase™ II Enzyme Mix (Thermo Fisher Scientific). Integrity of the expression cassette was confirmed by restriction analysis, and sequencing of promoter/gene/terminator region.

### Heterologous expression in yeast

The *S*. *cerevisiae* strains used in this study included double mutant strains DEY1453 (*fet3fet4*), ZHY3 (*zrt1zrt2*) and corresponding parental wildtype strain DY1457; and single mutant strain HomDip-YOL122C lacking SMF1 (transOMIC). Mutant and wild type strains were transformed with the yeast expression vector pDR195GTW containing one of the six cacao *TcNRAMP* genes including splice variant *TcNRAMP5s* or the empty vector pDR195 using a yeast transformation kit (Sigma-Aldrich) following manufacturer’s instructions. Transformants were selected on synthetic defined medium containing 6.7 g/l yeast nitrogen base without amino acids (Thermo Fisher Scientific), 1 g/l of amino acid supplement without uracil (Sigma Aldrich) and 2% glucose, designated as SD-U medium. The SD-U medium was supplemented with 100 μM of ferric chloride (FeCl_3_), zinc chloride (ZnCl_2_) and manganese sulfate (MnSO_4_) for the selection of DEY1453, ZHY3 and YOL122C transformants, respectively. For the complementation assay, a single yeast colony from each plate was inoculated into the liquid medium used in the selection and grown to an OD_600_ of 1.0. The yeast cells were pelleted by centrifugation, washed in sterile water to remove metal adsorbed to cell walls and diluted to an OD_600_ of 0.1. Four 10-fold serial dilutions were prepared in water and 5 μl were spotted on the plate. Transformed *fet3fet4* cells were spotted on SD-U medium plate (pH 4.0) supplemented with 10 μM FeCl_3_ or 10 μM Fe chelator BPS. Transformed *zrt1zrt2* cells were assessed on SD-U medium plate (pH 5.8) supplemented with 100 μM ZnCl_2_ or with 100 μM of the metal chelator Ethylenediaminetetraacetic acid (EDTA), 10 μM each of FeCl_3_ and ZnCl_2_.Transformed *smf1* cells were grown on SD-U medium plate (pH 5.2) supplemented with 100 μM MnSO_4_ or with 12.5 mM EGTA. The growth assay of ZHY3 and DEY1453 cells was conducted in liquid low zinc medium (LZM100)^7^ and low iron medium (LIM1)^45^, respectively. The wild type yeast strain DY1457 transformed with the empty vector pDR195 was included as a positive control in all three assays.

For the cadmium sensitivity assay, transformed wild type cells DY1457 were spotted on SD-U medium plate without cadmium chloride (CdCl_2_), and supplemented with 10 and 20 μM CdCl_2_. The plates were incubated at 30 °C for 3 (*fet3fet4*, *smf1*, WT) or 6 (*zrt1zrt2*) days before photography. For quantitative assessment of growth response to Cd^2+^, 10 ml of liquid SD-U medium, which contained 0, 2, 5, 10, 20 and 50 μM CdCl_2_, was inoculated with primary culture of the transformed DY1457 cells at an OD_600_ of 0.01. The cells were grown at 30 °C with shaking at 250 rpm for 72 h. The OD_600_ was measured on SpectraMax i3x (Molecular Devices) microplate reader. The growth inhibition was calculated by comparing final OD_600_ of the treated cultures with control (No CdCl_2_).

### Determination of Cd^2+^ accumulation

To determine Cd^2+^ accumulation in yeast, 10 ml of SD-U liquid culture supplemented with 2 μM CdCl_2_ was inoculated with pre-culture of transformed wild type (DY1457) cells at an initial OD_600_ of 0.I. The cells were cultured at 30 °C with shaking at 250 rpm for 72 h and centrifuged. The pelleted cells were washed with cold 20 mM EDTA for 10 min, rinsed three times with deionized water, and dried at 70 °C for 2 days. The dried cells were digested for 8 hours in 5 mL of 70% nitric acid (TraceSELECT™ grade) in closed glass vessels at 110 °C. All digestions were performed in duplicate, and for quality control, a blank and a plant certified reference material (IAEA-359 cabbage leaves) were included. The Cd^2+^accumulation in the cells was determined by inductively coupled plasma mass spectrometry (Thermo Scientific™ iCAP™ Q ICP-MS).

### Data analysis

Significance analysis was performed by Student’s t test using SPSS software. The difference at P < 0.05 and P < 0.01 was considered as significant and highly significant, respectively.

## Electronic supplementary material

Supplementary Information

## Data Availability

Sequence data from this article can be found in the GenBank database under the following accession numbers: *TcNRAMP1* (MH615045), *TcNRAMP1m* (MH615046), *TcNRAMP2* (MH615047), *TcNRAMP3* (MH615048), *TcNRAMP5* (MH615049), *TcNRAMP5s* (MH615050) and *TcNRAMP6* (MH615041).

## References

[1] Bozzi AT (2016). Conserved methionine dictates substrate preference in Nramp-family divalent metal transporters. Proc Natl Acad Sci.

[2] Bozzi AT (2016). Crystal structure and conformational change mechanism of a bacterial Nramp-family divalent metal transporter. Structure.

[3] Chavez E (2016). Evaluation of soil amendments as a remediation alternative for cadmium-contaminated soils under cacao plantations. Environ Sci Pollut Res.

[4] Yang Y (2018). Can liming reduce cadmium (Cd) accumulation in rice (*Oryza sativa*) in slightly acidic soils? A contradictory dynamic equilibrium between Cd uptake capacity of roots and Cd immobilisation in soils. Chemosphere.

[5] Tang L (2017). Knockout of *OsNramp5* using the CRISPR/Cas9 system produces low Cd-accumulating indica rice without compromising yield. Sci Rep.

[6] Dix DR, Bridgham JT, Broderius MA, Byersdorfer CA, Eide DJ (1994). The *FET4* gene encodes the low affinity Fe(II) transport protein of *Saccharomyces cerevisiae*. J Biol Chem.

[7] Zhao H, Eide D (1996). The *ZRT2* gene encodes the low affinity zinc transporter in *Saccharomyces cerevisiae*. J Biol Chem.

[8] Thomine S, Wang R, Ward JM, Crawford NM, Schroeder JI (2000). Cadmium and iron transport by members of a plant metal transporter family in *Arabidopsis* with homology to *Nramp* genes. Proc Natl Acad Sci.

[9] Yang M (2014). OsNRAMP5 contributes to manganese translocation and distribution in rice shoots. J Exp Bot.

[10] Takahashi R (2011). The OsNRAMP1 iron transporter is involved in Cd accumulation in rice. J Exp Bot.

[11] Cailliatte R, Lapeyre B, Briat JF, Mari S, Curie C (2009). The NRAMP6 metal transporter contributes to cadmium toxicity. Biochem J.

[12] Qin L (2017). Genome-wide identification and expression analysis of *NRAMP* family genes in soybean (*Glycine Max* L.). Front Plant Sci.

[13] Cannon SB, Mitra A, Baumgarten A, Young ND, May G (2004). The roles of segmental and tandem gene duplication in the evolution of large gene families in *Arabidopsis thaliana*. BMC Plant Biol.

[14] Soltis DE, Visger CJ, Soltis PS (2014). The polyploidy revolution then … and now: Stebbins revisited. Am J Bot.

[15] Bowers JL, Chapman BA, Rong J, Paterson AH (2003). Unraveling angiosperms genome evolution by phylogenetic analysis of chromosomal duplications events. Nature.

[16] Argout X (2011). The genome of *Theobroma cacao*. Nat Genet.

[17] Maser P (2001). Phylogenetic relationships within cation transporter families of *Arabidopsis*. Plant Physiol.

[18] Force A (1999). Preservation of duplicate genes by complementary, degenerative mutations. Genetics.

[19] Curie C, Alonso JM, Le Jean M, Ecker JR, Briat JF (2000). Involvement of NRAMP1 from *Arabidopsis thaliana* in iron transport. Biochem J.

[20] Cailliatte R, Schikora A, Briat JF, Mari S, Curie C (2010). High-affinity manganese uptake by the metal transporter NRAMP1 is essential for *Arabidopsis* growth in low manganese conditions. Plant Cell.

[21] Sasaki A, Yamaji N, Yokosho K, Ma JF (2012). Nramp5 is a major transporter responsible for manganese and cadmium uptake in rice. Plant Cell.

[22] Gao H (2018). NRAMP2, a trans-Golgi network-localized manganese transporter, is required for *Arabidopsis* root growth under manganese deficiency. New Phytol.

[23] Fujimaki S (2010). Tracing cadmium from culture to spikelet: Noninvasive imaging and quantitative characterization of absorption, transport, and accumulation of cadmium in an intact rice plant. Plant Physiol.

[24] Boyd RS (2012). Plant defense using toxic inorganic ions: conceptual models of the defensive enhancement and joint effects hypotheses. Plant Sci.

[25] Hörger AC, Fones HN, Preston GM (2013). The current status of the elemental defense hypothesis in relation to pathogens. Front Plant Sci.

[26] Poschenrieder C, Cabot C, Martos S, Gallego B, Barceló J (2013). Do toxic ions induce hormesis in plants?. Plant Sci.

[27] Chouhan S, Verma SC, Thakur M (2017). Effect of cadmium on biology of tobacco caterpillar *Spodoptera litura* fabricius (lepidoptera: Noctuidae). Nat Environ Pollut Technol.

[28] Kazemi-Dinan A, Thomaschky S, Stein RJ, Krämer U, Müller C (2014). Zinc and cadmium hyperaccumulation act as deterrents towards specialist herbivores and impede the performance of a generalist herbivore. New Phytol.

[29] Li K (2018). Effects of Cd accumulation on cutworm *Spodoptera litura* larvae via Cd treated Chinese flowering cabbage *Brassica campestris* and artificial diets. Chemosphere.

[30] Plaza S (2015). Wounding of *Arabidopsis halleri* leaves enhances cadmium accumulation that acts as a defense against herbivory. Biometals.

[31] Vlahović M (2017). Influence of dietary cadmium exposure on fitness traits and its accumulation (with an overview on trace elements) in *Lymantria dispar* larvae. Comp Biochem Physiol C Toxicol Pharmacol.

[32] Zhan H (2018). Effects of Cd^2+^ exposure on key life history traits and activities of four metabolic enzymes in *Helicoverpa armigera* (Lepidopteran: Noctuidae). Chem Ecol.

[33] Oomen RJFJ (2009). Functional characterization of NRAMP3 and NRAMP4 from the metal hyperaccumulator *Thlaspi caerulescens*. New Phytol.

[34] Peng Fan, Wang Chao, Zhu Jianshu, Zeng Jian, Kang Houyang, Fan Xing, Sha Lina, Zhang Haiqin, Zhou Yonghong, Wang Yi (2018). Expression of TpNRAMP5, a metal transporter from Polish wheat (Triticum polonicum L.), enhances the accumulation of Cd, Co and Mn in transgenic Arabidopsis plants. Planta.

[35] Tang Z (2017). Allelic variation of *NtNramp5* associated with cultivar variation in cadmium accumulation in tobacco. Plant Cell Physiol.

[36] Wu D (2016). The HvNramp5 transporter mediates uptake of cadmium and manganese, but not iron. Plant Physiol.

[37] Ehrnstorfer IA, Geertsma ER, Pardon E, Steyaert J, Dutzler R (2014). Crystal structure of a SLC11 (NRAMP) transporter reveals the basis for transition-metal ion transport. Nat Struct Mol Biol.

[38] Pottier M (2015). Identification of mutations allowing Natural Resistance Associated Macrophage Proteins (NRAMP) to discriminate against cadmium. Plant J.

[39] Xia J, Yamaji N, Kasai T, Ma JF (2010). Plasma membrane-localized transporter for aluminum in rice. Proc Natl Acad Sci.

[40] Ishikawa S (2012). Ion-beam irradiation, gene identification, and marker-assisted breeding in the development of low-cadmium rice. Proc Natl Acad Sci.

[41] Mason AB, Allen KE, Slayman CW (2006). Effects of C-terminal truncations on trafficking of the yeast plasma membrane H^+^-ATPase. J Biol Chem.

[42] Edgar RC (2004). MUSCLE: Multiple sequence alignment with high accuracy and high throughput. Nucleic Acids Res..

[43] Kumar S, Stecher G, Tamura K (2016). MEGA7: Molecular Evolutionary Genetics Analysis Version 7.0 for bigger datasets.. Mol Biol Evol.

[44] Letunic I, Bork P (2016). Interactive tree of life (iTOL)v3: an online tool for the display and annotation of phylogenetic and other trees. Nucleic Acids Res.

[45] Eide D, Guarente L (1992). Increased dosage of a transcriptional activator gene enhances iron-limited growth of *Saccharomyces cerevisiae*. J Gen Microbiol.

