# Supplementary material for: Evolution, and functional analysis of Natural Resistance-Associated Macrophage Proteins (NRAMPs) from Theobroma cacao and their role in cadmium accumulation
Source: Sci Rep. 2018 Sep 26;8:14412. doi: 10.1038/s41598-018-32819-y (PMC6158261; doi:10.1038/s41598-018-32819-y)
Supplement: Supplementary file 1 — Supplementary Information [file 41598_2018_32819_MOESM1_ESM.docx]

**Evolution, and functional analysis of Natural Resistance-Associated Macrophage Proteins (NRAMPs) from *Theobroma cacao* and their role in cadmium accumulation**

Authors

Ihsan Ullah^1^, Yirong Wang^2^, David J. Eide^2^ & Jim M. Dunwell^1*^

^1^School of Agriculture, Policy and Development, University of Reading, Earley Gate, Reading RG6 6AR, UK

^2^Department of Nutritional Sciences, University of Wisconsin-Madison, Madison, WI 53706, USA

**Supplementary figures**

**
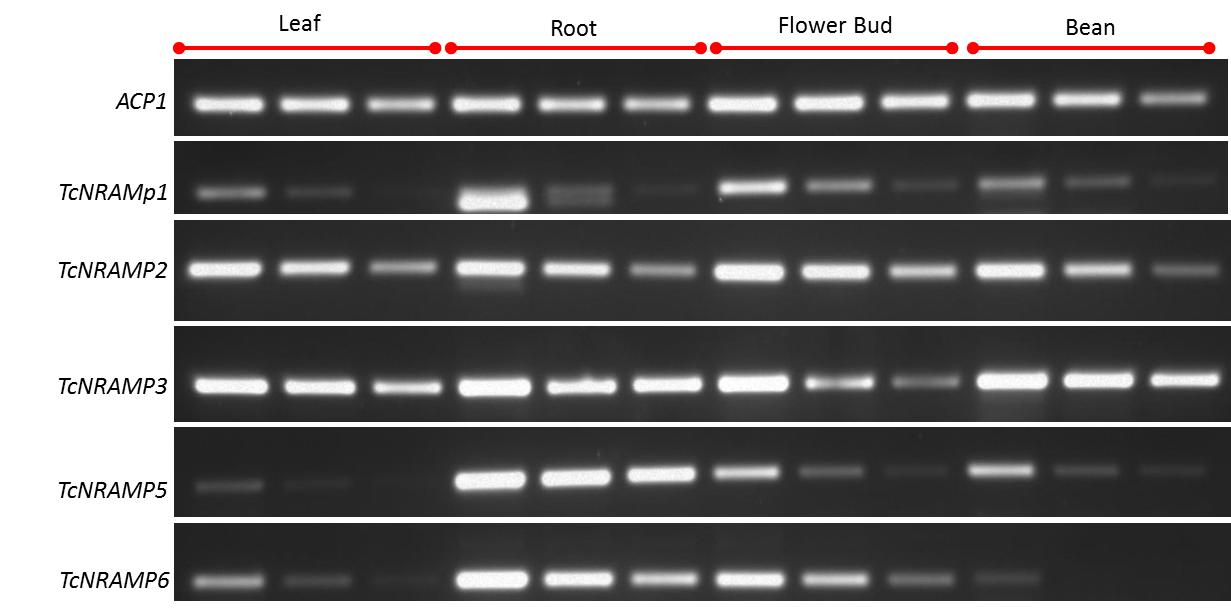
**

**Supplementary Figure S1**. **Expression analyses of Cacao *NRAMP* genes*.*** Gel image of RT-PCR products of *TcNRAMP1, 2,* *3, 5* and *6* amplified from leaf, root, unopened flower bud and beans. Acyl Carrier Protein (*ACP1*) was incorporated as reference gene. RT-PCR was performed using three serial dilutions (5-fold) of template cDNA (from left to right) from each tissue.


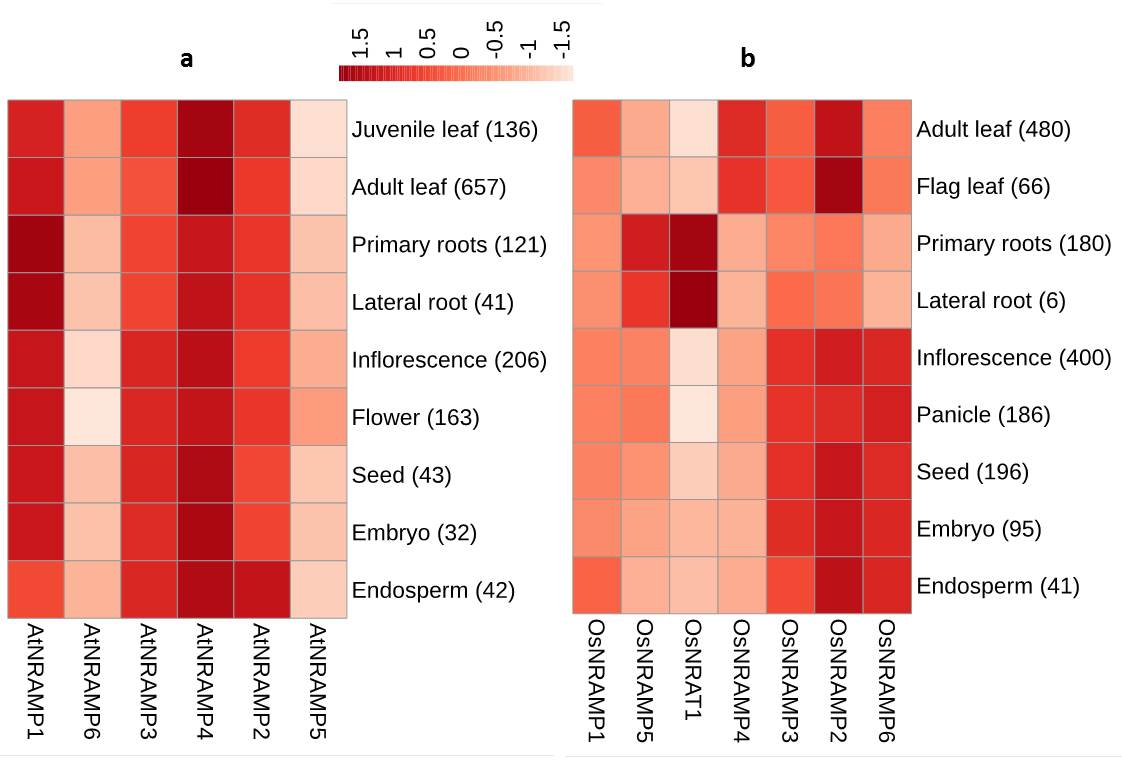


(a)

(b)

**Supplementary Figure S2. Heatmap illustration of expression of *NRAMP* genes across different tissue types.** (a) Gene expression pattern of *Arabidopsis* *NRAMP* genes (b) Gene expression pattern of rice *NRAMP* genes. The data were retrieved from “Affymetrix *Arabidopsis* ATH1 Genome Array” and “Affymetrix Rice Genome Array” platforms at the Genevestigator database (<https://genevestigator.com>). Log2 based values were used to generate the heatmap using ClustVis web tool (<http://biit.cs.ut.ee/clustvis/>). The colour scale shown at the top depicts the relative expression level.


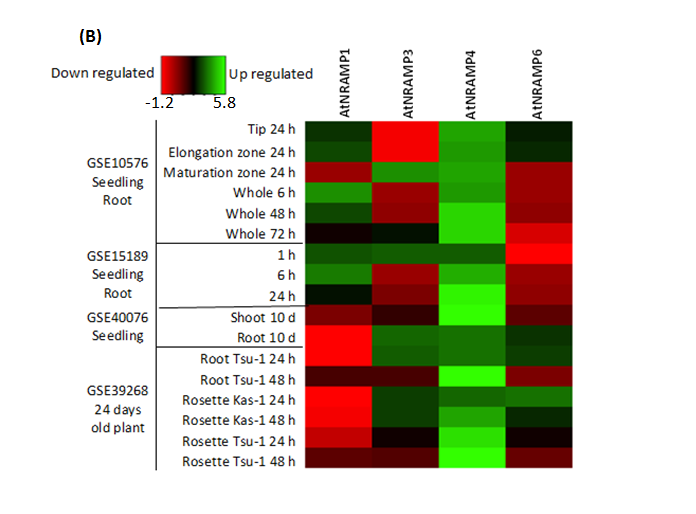


**Supplementary Figure S3**. **Heatmap illustration of expression of *Arabidopsis* cadmium transporters in conditions of iron deficiency.** Fold change in expression of *Arabidopsis* NRAMP cadmium transporters in response to iron deficient conditions. The data were retrieved from “Affymetrix *Arabidopsis* ATH1 Genome Array” platform at the Genevestigator database (<https://genevestigator.com>).


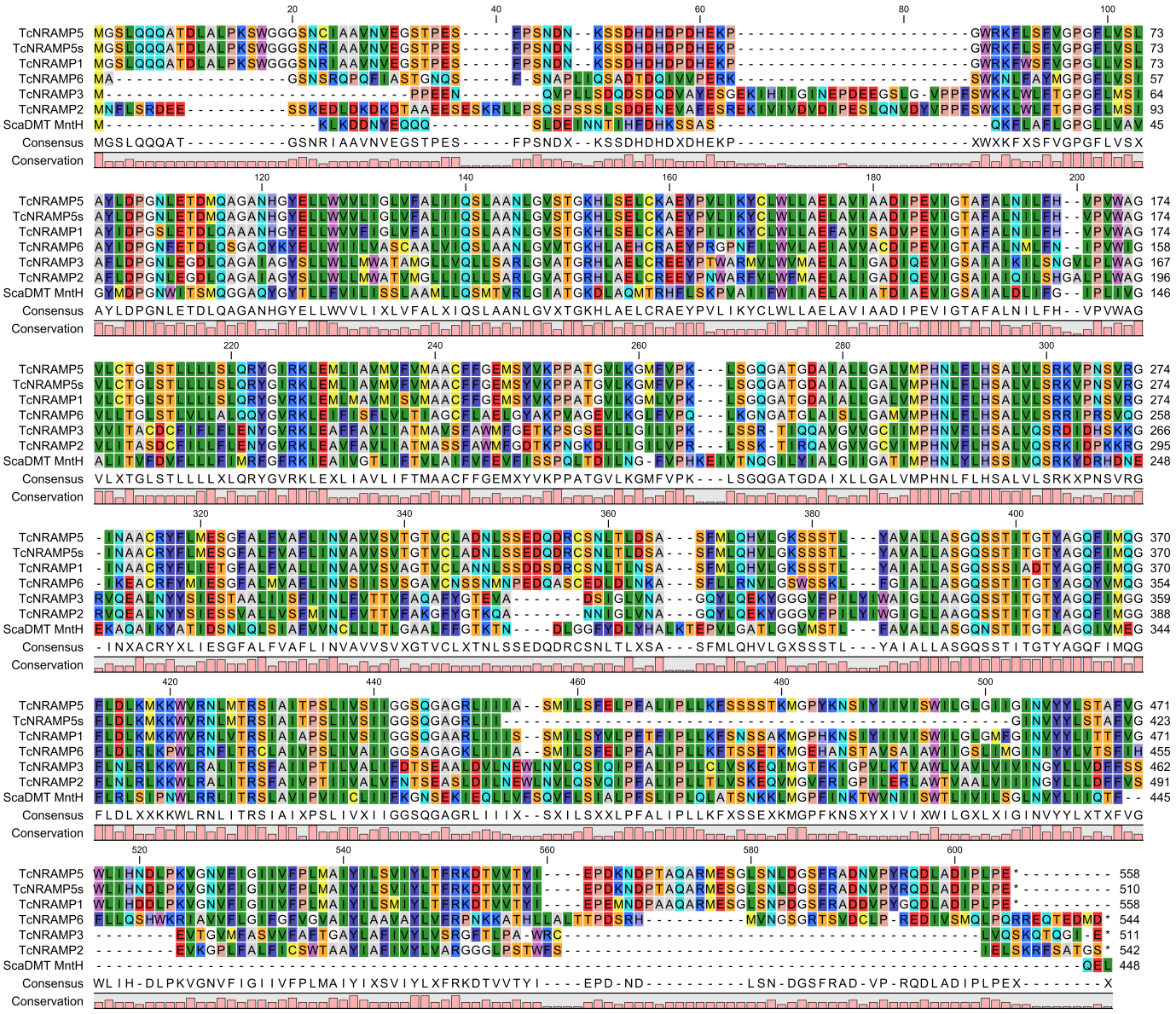

**Supplementary Figure S4. Amino acid sequences and the predicted transmembrane structure of cacao (Tc) NRAMPs.** (a) Amino acid sequence alignment of TcNRAMP1, 2, 3, 5, 6, splice variant of TcNRAMP5 (TcNRAMP5s) from cacao clone NA702, and divalent metal cation transporter (ScaDMT MntH; GenBank: WP_002435436.1) from *Staphylococcus capitis*. Arrows indicate conserved residues reportedly involved in metal selectivity^1,29^. (b and c) Prediction of the transmembrane structure of TcNRAMP5 and TcNRAMP5s, respectively, using the PROTTER online tool. (<http://wlab.ethz.ch/protter/>).

**(c)**

**(b)**

**(a)**


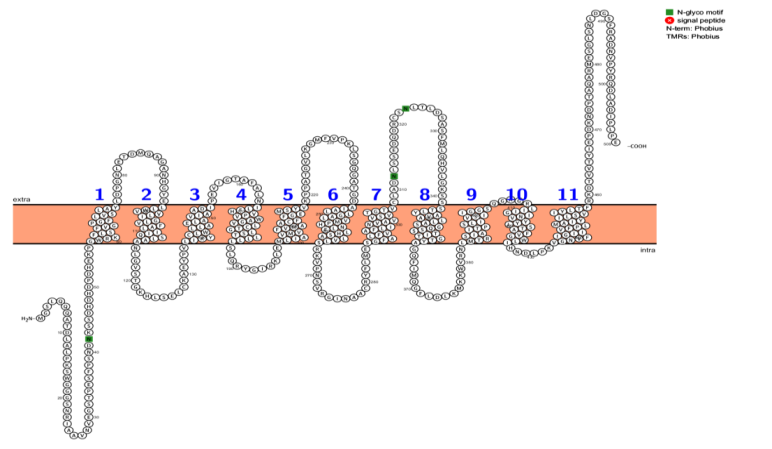


**Supplementary Tables**

**Supplementary Table S1.** Physio-chemical properties of NRAMP homologs found in cacao.

| **Designation** | **Protein ID** | **Length**  **(aa)** | **Isoelectric**  **point** | **MW**  **(kDa)** | **Probability of N-in** | **TMD/terminus** |
| --- | --- | --- | --- | --- | --- | --- |
| TcNRAMP1 | XP_007019142 | 561 | 7.06 | 60.68 | 0.10 | 13/OUT **>** IN |
| TcNRAMP2 | XP_007018563 | 541 | 5.62 | 59.43 | 0.68 | 11/OUT **>** IN |
| TcNRAMP3 | XP_007047433 | 510 | 4.98 | 55.71 | 0.13 | 11/OUT **>** IN |
| TcNRAMP5 | XP_007019147 | 557 | 6.37 | 60.18 | 0.83 | 12/IN **>** IN |
| TcNRAMP6 | XP_007023419 | 543 | 8.29 | 58.86 | 0.98 | 12/IN **>** IN |

**Supplementary Table S2.** List of 170 NRAMP homologs from two algae and 28 plant species, and a bacterium (*Paenibacillus pini* ) manganese transporter MnhT. Names of the species are abbreviated with a two/three-letter code. Number following NRAMP represents multiple members within a single species. NRAMP numbering is arbitrary except for rice and *Arabidopsis*.

| **Phytozome ID** | **Designated**  **Name** | **Species** | **Family** | | **Description** | |
| --- | --- | --- | --- | --- | --- | --- |
| Aquca_014_00265 | AquNRAMP1 | *Aquilegia coerulea* | Ranunculaceae | | PTHR11706:SF38 - manganese transporter | |
| Aquca_014_00519 | AquNRAMP2 | *Aquilegia coerulea* | Ranunculaceae | | PTHR11706:SF38 - manganese transporter | |
| Aquca_042_00040 | AquNRAMP3 | *Aquilegia coerulea* | Ranunculaceae | | PTHR11706:SF46 - metal transporter NRAMP2-related | |
| AT1G80830 | AtNRAMP1 | *Arabidopsis thaliana* | Brassicaceae | | metal transporter NRAMP1 | |
| AT1G47240 | AtNRAMP2 | *Arabidopsis thaliana* | Brassicaceae | | NRAMP metal ion transporter2 | |
| AT2G23150 | AtNRAMP3 | *Arabidopsis thaliana* | Brassicaceae | | metal transporter NRAMP3 | |
| AT5G67330 | AtNRAMP4 | *Arabidopsis thaliana* | Brassicaceae | | metal transporter NRAMP4 | |
| AT4G18790 | AtNRAMP5 | *Arabidopsis thaliana* | Brassicaceae | | NRAMP metal ion transporter family protein | |
| AT1G15960 | AtNRAMP6 | *Arabidopsis thaliana* | Brassicaceae | | NRAMP metal ion transporter6 | |
| evm_27.model.AmTr_v1.0_  scaffold00001.588 | AtrNRAMP1 | *Amborella trichopoda* | Amborellaceae | | PTHR11706:SF46 - metal transporter NRAMP2-related | |
| evm_27.model.AmTr_v1.0_  scaffold00092.96 | AtrNRAMP2 | *Amborella trichopoda* | Amborellaceae | | PTHR11706:SF33 - metal transporter NRAMP1 | |
| evm_27.model.AmTr_v1.0_  scaffold00203.5 | AtrNRAMP3 | *Amborella trichopoda* | Amborellaceae | | Mn2+ and Fe2+ transporters of the NRAMP family | |
| Brara.B02391 | BrNRAMP1 | *Brassica rapa* | Brassicaceae | | PTHR11706:SF33 - metal transporter NRAMP | |
| Brara.C02490 | BrNRAMP2 | *Brassica rapa* | Brassicaceae | | PTHR11706:SF46 - metal transporter NRAMP2-related | |
| Brara.F01109 | BrNRAMP3 | *Brassica rapa* | Brassicaceae | | PTHR11706:SF33 - metal transporter NRAMP | |
| Brara.G01168 | BrNRAMP4 | *Brassica rapa* | Brassicaceae | | PTHR11706:SF8 - metal transporter NRAMP1 homolog | |
| Brara.G03697 | BrNRAMP5 | *Brassica rapa* | Brassicaceae | | PTHR11706:SF33 - metal transporter NRAMP | |
| Brara.H00421 | BrNRAMP6 | *Brassica rapa* | Brassicaceae | | PTHR11706:SF46 - metal transporter NRAMP2-related | |
| Brara.I04545 | BrNRAMP7 | *Brassica rapa* | Brassicaceae | | PTHR11706:SF46 - metal transporter NRAMP2-related | |
| Brara.K00425 | BrNRAMP8 | *Brassica rapa* | Brassicaceae | | PTHR11706:SF46 - metal transporter NRAMP2-related | |
| Bostr.0568s0157 | BsNRAMP1 | *Boechera stricta* | Brassicaceae | | PTHR11706:SF8 - metal transporter NRAMP1 homolog | |
| **Phytozome ID** | **Designated**  **Name** | **Species** | **Family** | | **Description** | |
| Bostr.12302s0150 | BsNRAMP2 | *Boechera stricta* | Brassicaceae | | PTHR11706:SF46 - metal transporter NRAMP2-related | |
| Bostr.27895s0165 | BsNRAMP3 | *Boechera stricta* | Brassicaceae | | PTHR11706:SF46 - metal transporter NRAMP2-related | |
| Bostr.30275s0354 | BsNRAMP4 | *Boechera stricta* | Brassicaceae | | PTHR11706:SF46 - metal transporter NRAMP2-related | |
| Bostr.7128s0215 | BsNRAMP5 | *Boechera stricta* | Brassicaceae | | PTHR11706:SF33 - metal transporter NRAMP | |
| Brast01G259200 | BstNRAMP1 | *Brachypodium stacei* | Poaceae | | PTHR11706:SF47 - manganese transporter | |
| Brast02G087200 | BstNRAMP2 | *Brachypodium stacei* | Poaceae | | PTHR11706:SF51 - manganese transporter | |
| Brast02G231000 | BstNRAMP3 | *Brachypodium stacei* | Poaceae | | PTHR11706:SF8 - metal transporter NRAMP1 homolog | |
| Brast06G158000 | BstNRAMP4 | *Brachypodium stacei* | Poaceae | | PTHR11706:SF38 - manganese transporter | |
| Brast07G209500 | BstNRAMP5 | *Brachypodium stacei* | Poaceae | | PTHR11706:SF33 - metal transporter NRAMP | |
| Brast10G035600 | BstNRAMP6 | *Brachypodium stacei* | Poaceae | | PTHR11706:SF46 - metal transporter NRAMP2-related | |
| Carubv10004570m | CrNRAMP1 | *Capsella rubella* | Brassicaceae | | PTHR11706:SF46 - metal transporter NRAMP2-related | |
| Carubv10008822m | CrNRAMP2 | *Capsella rubella* | Brassicaceae | | PTHR11706:SF46 - metal transporter NRAMP2-related | |
| Carubv10008854m | CrNRAMP3 | *Capsella rubella* | Brassicaceae | | PTHR11706:SF33 - metal transporter NRAMP | |
| Carubv10021908m | CrNRAMP4 | *Capsella rubella* | Brassicaceae | | PTHR11706:SF33 - metal transporter NRAMP | |
| Carubv10022990m | CrNRAMP5 | *Capsella rubella* | Brassicaceae | | PTHR11706:SF46 - metal transporter NRAMP2-related | |
| Carubv10026252m | CrNRAMP6 | *Capsella rubella* | Brassicaceae | | PTHR11706:SF8 - metal transporter NRAMP1 homolog | |
| Cre05.g248300 | CrsNRAMP1 | *Chlamydomonas reinhardtii* | Chlamydomonas | | PTHR11706:SF8 - metal transporter NRAMP1 homolog | |
| Cre17.g707700 | CrsNRAMP2 | *Chlamydomonas reinhardtii* | Chlamydomonas | | Manganese/metal transporter, NRAMP homolog | |
| Cucsa.060540 | CsaNRAMP1 | *Cucumis sativus* | Cucurbitaceae | | PTHR11706:SF38 - manganese transporter | |
| Cucsa.073010 | CsaNRAMP2 | *Cucumis sativus* | Cucurbitaceae | | PTHR11706:SF46 - metal transporter NRAMP2-related | |
| Cucsa.177200 | CsaNRAMP3 | *Cucumis sativus* | Cucurbitaceae | | PTHR11706:SF33 - metal transporter NRAMP1 | |
| Cucsa.177590 | CsaNRAMP4 | *Cucumis sativus* | Cucurbitaceae | | PTHR11706:SF33 - metal transporter NRAMP1 | |
| orange1.1g008955 | CsNRAMP1 | *Citrus sinensis* | Theaceae | | PTHR11706:SF33 - metal transporter NRAMP1 | |
| orange1.1g009128 | CsNRAMP2 | *Citrus sinensis* | Theaceae | | PTHR11706:SF33 - metal transporter NRAMP1 | |
| orange1.1g009526 | CsNRAMP3 | *Citrus sinensis* | Theaceae | | PTHR11706:SF46 - metal transporter NRAMP2-related | |
| orange1.1g010382 | CsNRAMP4 | *Citrus sinensis* | Theaceae | | PTHR11706:SF46 - metal transporter NRAMP2-related | |
| **Phytozome ID** | **Designated**  **Name** | **Species** | **Family** | | **Description** | |
| orange1.1g012869 | CsNRAMP5 | *Citrus sinensis* | Theaceae | | PTHR11706:SF46 - metal transporter NRAMP2-related | |
| orange1.1g013769 | CsNRAMP6 | *Citrus sinensis* | Theaceae | | PTHR11706:SF33 - metal transporter NRAMP1 | |
| Eucgr.F00133 | EgNRAMP1 | *Eucalyptus grandis* | Myrtaceae | | PTHR11706:SF46 - metal transporter NRAMP2-related | |
| Eucgr.F02273 | EgNRAMP2 | *Eucalyptus grandis* | Myrtaceae | | PTHR11706:SF46 - metal transporter NRAMP2-related | |
| Eucgr.F04336 | EgNRAMP3 | *Eucalyptus grandis* | Myrtaceae | | PTHR11706:SF38 - manganese transporter | |
| Eucgr.H02266 | EgNRAMP4 | *Eucalyptus grandis* | Myrtaceae | | PTHR11706:SF46 - metal transporter NRAMP2-related | |
| Eucgr.I00738 | EgNRAMP5 | *Eucalyptus grandis* | Myrtaceae | | PTHR11706:SF46 - metal transporter NRAMP2-related | |
| Eucgr.J03115 | EgNRAMP6 | *Eucalyptus grandis* | Myrtaceae | | PTHR11706:SF33 - metal transporter NRAMP1 | |
| Eucgr.L02462 | EgNRAMP7 | *Eucalyptus grandis* | Myrtaceae | | PTHR11706:SF38 - manganese transporter | |
| Glyma.01G190700 | GmNRAMP1 | *Glycine max* | Fabaceae | | PTHR11706:SF46 - metal transporter NRAMP2-related | |
| Glyma.13G369900 | GmNRAMP10 | *Glycine max* | Fabaceae | | PTHR11706:SF33 - metal transporter NRAMP1 | |
| Glyma.15G003500 | GmNRAMP11 | *Glycine max* | Fabaceae | | PTHR11706:SF33 - metal transporter NRAMP1 | |
| Glyma.16G027800 | GmNRAMP12 | *Glycine max* | Fabaceae | | PTHR11706:SF46 - metal transporter NRAMP2-related | |
| Glyma.17G165200 | GmNRAMP13 | *Glycine max* | Fabaceae | | PTHR11706:SF46 - metal transporter NRAMP2-related | |
| Glyma.04G044000 | GmNRAMP2 | *Glycine max* | Fabaceae | | PTHR11706:SF46 - metal transporter NRAMP2-related | |
| Glyma.05G101700 | GmNRAMP3 | *Glycine max* | Fabaceae | | PTHR11706:SF46 - metal transporter NRAMP2-related | |
| Glyma.06G044200 | GmNRAMP4 | *Glycine max* | Fabaceae | | PTHR11706:SF46 - metal transporter NRAMP2-related | |
| Glyma.06G115800 | GmNRAMP5 | *Glycine max* | Fabaceae | | PTHR11706:SF38 - manganese transporter | |
| Glyma.07G023600 | GmNRAMP6 | *Glycine max* | Fabaceae | | PTHR11706:SF33 - metal transporter NRAMP1 | |
| Glyma.07G058900 | GmNRAMP7 | *Glycine max* | Fabaceae | | PTHR11706:SF46 - metal transporter NRAMP2-related | |
| Glyma.08G218200 | GmNRAMP8 | *Glycine max* | Fabaceae | | PTHR11706:SF33 - metal transporter NRAMP1 | |
| Glyma.11G051500 | GmNRAMP9 | *Glycine max* | Fabaceae | | PTHR11706:SF46 - metal transporter NRAMP2-related | |
| Gorai.003G027800 | GrNRAMP1 | *Gossypium raimondii* | Malvaceae | | PTHR11706:SF46 - metal transporter NRAMP2-related | |
| Gorai.007G219700 | GrNRAMP2 | *Gossypium raimondii* | Malvaceae | | PTHR11706:SF46 - metal transporter NRAMP2-related | |
| Gorai.009G124200 | GrNRAMP3 | *Gossypium raimondii* | Malvaceae | | PTHR11706:SF33 - metal transporter NRAMP1 | |
| Gorai.009G217100 | GrNRAMP4 | *Gossypium raimondii* | Malvaceae | | PTHR11706:SF46 - metal transporter NRAMP2-related | |
| Gorai.009G248100 | GrNRAMP5 | *Gossypium raimondii* | Malvaceae | | PTHR11706:SF38 - manganese transporter | |
| Gorai.010G052500 | GrNRAMP6 | *Gossypium raimondii* | Malvaceae | | PTHR11706:SF38 - manganese transporter | |
| **Phytozome ID** | **Designated**  **Name** | **Species** | **Family** | | **Description** | |
| Lus10004317 | LuNRAMP1 | *Linum usitatissimum* | Linaceae | | PTHR11706:SF46 - metal transporter NRAMP2-related | |
| Lus10010395 | LuNRAMP2 | *Linum usitatissimum* | Linaceae | | PTHR11706:SF46 - metal transporter NRAMP2-related | |
| Lus10014868 | LuNRAMP3 | *Linum usitatissimum* | Linaceae | | PTHR11706:SF46 - metal transporter NRAMP2-related | |
| Lus10018132 | LuNRAMP4 | *Linum usitatissimum* | Linaceae | | PTHR11706:SF38 - manganese transporter | |
| Lus10024069 | LuNRAMP5 | *Linum usitatissimum* | Linaceae | | PTHR11706:SF33 - metal transporter NRAMP1 | |
| Lus10028542 | LuNRAMP6 | *Linum usitatissimum* | Linaceae | | PTHR11706:SF38 - manganese transporter | |
| Lus10032853 | LuNRAMP7 | *Linum usitatissimum* | Linaceae | | PTHR11706:SF46 - metal transporter NRAMP2-related | |
| Lus10041652 | LuNRAMP8 | *Linum usitatissimum* | Linaceae | | PTHR11706:SF33 - metal transporter NRAMP1 | |
| GSMUA_AchrUn_  randomT11650 | MaNRAMP1 | *Musa acuminata* | Musaceae | | PTHR11706:SF38 - manganese transporter | |
| GSMUA_AchrUn_  randomT21120 | MaNRAMP2 | *Musa acuminata* | Musaceae | | PTHR11706:SF33 - metal transporter NRAMP1 | |
| GSMUA_Achr6G07820 | MaNRAMP3 | *Musa acuminata* | Musaceae | | PTHR11706:SF51 - MANGANESE TRANSPORTE | |
| GSMUA_Achr4G32750 | MaNRAMP4 | *Musa acuminata* | Musaceae | | PTHR11706:SF38 - manganese transporter | |
| GSMUA_Achr11G26320 | MaNRAMP5 | *Musa acuminata* | Musaceae | | PTHR11706:SF38 - manganese transporter | |
| Medtr2g104990 | MtNRAMP1 | *Medicago truncatula* | Fabaceae | | PTHR11706:SF33 - metal transporter NRAMP1 | |
| Medtr3g088440 | MtNRAMP2 | *Medicago truncatula* | Fabaceae | | PTHR11706:SF38 - manganese transporter | |
| Medtr3g088460 | MtNRAMP3 | *Medicago truncatula* | Fabaceae | | PTHR11706:SF38 - manganese transporter | |
| Medtr3g102620 | MtNRAMP4 | *Medicago truncatula* | Fabaceae | | PTHR11706:SF46 - metal transporter NRAMP2-related | |
| Medtr4g095075 | MtNRAMP5 | *Medicago truncatula* | Fabaceae | | PTHR11706:SF46 - metal transporter NRAMP2-related | |
| Medtr5g016270 | MtNRAMP6 | *Medicago truncatula* | Fabaceae | | PTHR11706:SF46 - metal transporter NRAMP2-related | |
| Medtr8g028050 | MtNRAMP7 | *Medicago truncatula* | Fabaceae | | PTHR11706:SF46 - metal transporter NRAMP2-related | |
| LOC_Os07g15460 | OsNRAMP1 | *Oryza sativa* | Poaceae | | metal transporter Nramp6, putative, expressed | |
| LOC_Os03g11010 | OsNRAMP2 | *Oryza sativa* | Poaceae | | metal transporter NRAMP , putative, expressed | |
| LOC_Os06g46310 | OsNRAMP3 | *Oryza sativa* | Poaceae | | metal transporter Nramp6, putative, expressed | |
| LOC_Os01g31870 | OsNRAMP4 | *Oryza sativa* | Poaceae | | metal transporter NRAMP, putative, expressed | |
| LOC_Os07g15370 | OsNRAMP5 | *Oryza sativa* | Poaceae | | metal transporter Nramp6, putative, expressed | |
| LOC_Os12g39180 | OsNRAMP6 | *Oryza sativa* | Poaceae | | metal transporter NRAMP, putative, expressed | |
| **Phytozome ID** | **Designated**  **Name** | **Species** | **Family** | | **Description** | |
| LOC_Os02g03900 | OsNRAT1 | *Oryza sativa* | Poaceae | | Metal transporter Nramp6, putative, expressed | |
| Pp3c1_10690 | PpNRAMP1 | *Physcomitrella patens* | Funariaceae | | metal transporter NRAMP1 homolog | |
| Pp3c2_37310 | PpNRAMP2 | *Physcomitrella patens* | Funariaceae | | PTHR11706:SF8 - metal transporter NRAMP1 homolog | |
| Pp3c7_20820 | PpNRAMP3 | *Physcomitrella patens* | Funariaceae | | PTHR11706:SF8 - metal transporter NRAMP1 homolog | |
| Potri.001G044900 | PtNRAMP1 | *Populus trichocarpa* | Salicaceae | | similar to Metal transporter Nramp1 | |
| Potri.002G080400 | PtNRAMP2 | *Populus trichocarpa* | Salicaceae | | PTHR11706:SF38 - manganese transporter | |
| Potri.002G080500 | PtNRAMP3 | *Populus trichocarpa* | Salicaceae | | PTHR11706:SF38 - manganese transporter | |
| Potri.002G121000 | PtNRAMP4 | *Populus trichocarpa* | Salicaceae | | PTHR11706:SF46 - metal transporter NRAMP2-related | |
| Potri.007G050600 | PtNRAMP5 | *Populus trichocarpa* | Salicaceae | | PTHR11706:SF38 - manganese transporter | |
| Potri.007G050700 | PtNRAMP6 | *Populus trichocarpa* | Salicaceae | | similar to Metal transporter Nramp3 (AtNramp3) | |
| Pavir.Aa03191 | PvNRAMP1 | *Panicum virgatum* | Poaceae | | PTHR11706:SF48 - manganese transporter | |
| Pavir.Ib03924 | PvNRAMP10 | *Panicum virgatum* | Poaceae | | PTHR11706:SF46 - metal transporter NRAMP2-related | |
| Pavir.Ab00266 | PvNRAMP2 | *Panicum virgatum* | Poaceae | | PTHR11706:SF48 - manganese transporter | |
| Pavir.Ba03451 | PvNRAMP3 | *Panicum virgatum* | Poaceae | | PTHR11706:SF38 - manganese transporter | |
| Pavir.Ca02517 | PvNRAMP4 | *Panicum virgatum* | Poaceae | | PTHR11706:SF8 - metal transporter NRAMP1 homolog | |
| Pavir.Cb00495 | PvNRAMP5 | *Panicum virgatum* | Poaceae | | PTHR11706:SF46 - metal transporter NRAMP2-related | |
| Pavir.Da00057 | PvNRAMP6 | *Panicum virgatum* | Poaceae | | PTHR11706:SF33 - metal transporter NRAMP1 | |
| Pavir.Db00400 | PvNRAMP7 | *Panicum virgatum* | Poaceae | | PTHR11706:SF33 - metal transporter NRAMP1 | |
| Pavir.Ga02349 | PvNRAMP8 | *Panicum virgatum* | Poaceae | | PTHR11706:SF47 - manganese transporter | |
| Pavir.Ia01309 | PvNRAMP9 | *Panicum virgatum* | Poaceae | | PTHR11706:SF46 - metal transporter NRAMP2-related | |
| Sobic.001G170000 | SbNRAMP1 | *Sorghum bicolor* | Poaceae | | PTHR11706:SF46 - metal transporter NRAMP2-related | |
| Sobic.001G462500 | SbNRAMP2 | *Sorghum bicolor* | Poaceae | | similar to Integral membrane protein | |
| Sobic.002G095400 | SbNRAMP3 | *Sorghum bicolor* | Poaceae | | similar to Putative uncharacterized protein | |
| Sobic.002G095600 | SbNRAMP4 | *Sorghum bicolor* | Poaceae | | similar to OsNramp1 | |
| Sobic.004G029900 | SbNRAMP5 | *Sorghum bicolor* | Poaceae | | similar to Putative uncharacterized protein | |
| Sobic.005G134600 | SbNRAMP6 | *Sorghum bicolor* | Poaceae | | PTHR11706:SF47 - manganese transporter | |
| Sobic.008G143700 | SbNRAMP7 | *Sorghum bicolor* | Poaceae | | similar to Metal transporter Nramp2, putative, expressed | |
| Sobic.010G227800 | SbNRAMP8 | *Sorghum bicolor* | Poaceae | | similar to Metal transporter Nramp2, putative, expressed | |
| **Phytozome ID** | **Designated**  **Name** | **Species** | **Family** | | **Description** | |
| Sphfalx0000s0061 | SfNRAMP1 | *Sphagnum fallax* | Sphagnaceae | | PTHR11706:SF33 - metal transporter NRAMP1 | |
| Sphfalx0011s0196 | SfNRAMP2 | *Sphagnum fallax* | Sphagnaceae | | PTHR11706:SF33 - metal transporter NRAMP1 | |
| Sphfalx0225s0038 | SfNRAMP3 | *Sphagnum fallax* | Sphagnaceae | | PTHR11706:SF8 - metal transporter NRAMP1 homolog | |
| Sphfalx0287s0005 | SfNRAMP4 | *Sphagnum fallax* | Sphagnaceae | | PTHR11706:SF8 - metal transporter NRAMP1 homolog | |
| Seita.1G098100 | SiNRAMP1 | *Setaria italica* | Poaceae | | PTHR11706:SF48 - manganese transporter | |
| Seita.2G100300 | SiNRAMP2 | *Setaria italica* | Poaceae | | PTHR11706:SF38 - manganese transporter | |
| Seita.2G100400 | SiNRAMP3 | *Setaria italica* | Poaceae | | PTHR11706:SF41 - manganese transporter | |
| Seita.3G367300 | SiNRAMP4 | *Setaria italica* | Poaceae | | PTHR11706:SF46 - metal transporter NRAMP2-related | |
| Seita.7G056800 | SiNRAMP5 | *Setaria italica* | Poaceae | | PTHR11706:SF47 - manganese transporter | |
| Seita.9G171400 | SiNRAMP6 | *Setaria italica* | Poaceae | | PTHR11706:SF46 - metal transporter NRAMP2-related | |
| Seita.9G497200 | SiNRAMP7 | *Setaria italica* | Poaceae | | PTHR11706:SF51 - manganese transporter | |
| Solyc03g116900 | SlNRAMP1 | *Solanum lycopersicum* | Solanaceae | | mntH, metal transporter NRAMP | |
| Solyc04g078250 | SlNRAMP2 | *Solanum lycopersicum* | Solanaceae | | mntH, metal transporter NRAMP | |
| Solyc11g018530 | SlNRAMP3 | *Solanum lycopersicum* | Solanaceae | | mntH, metal transporter NRAMP | |
| Solyc02g092800 | SlNRAMP4 | *Solanum lycopersicum* | Solanaceae | | mntH, metal transporter NRAMP | |
| Solyc03g116900 | SlNRAMP5 | *Solanum lycopersicum* | Solanaceae | | mntH, metal transporter NRAMP | |
| 85390 | SmNRAMP1 | *Selaginella moellendorffii* | Selaginellaceae | | PTHR11706:SF38 - manganese transporter | |
| 86387 | SmNRAMP2 | *Selaginella moellendorffii* | Selaginellaceae | | PTHR11706:SF38 - manganese transporter | |
| 122391 | SmNRAMP3 | *Selaginella moellendorffii* | Selaginellaceae | | PTHR11706:SF38 - manganese transporter | |
| 173368 | SmNRAMP4 | *Selaginella moellendorffii* | Selaginellaceae | | PTHR11706:SF38 - manganese transporter | |
| 407690 | SmNRAMP5 | *Selaginella moellendorffii* | Selaginellaceae | | PTHR11706:SF38 - manganese transporter | |
| 417169 | SmNRAMP6 | *Selaginella moellendorffii* | Selaginellaceae | | PTHR11706:SF8 - metal transporter NRAMP1 homolog | |
| Spipo2G0094400 | SpNRAMP1 | *Spirodela polyrhiza* | Araceae | | Natural resistance-associated macrophage protein, putative | |
| **Phytozome ID** | **Designated**  **Name** | **Species** | **Family** | **Description** | |  |
| Spipo8G0025200 | SpNRAMP2 | *Spirodela polyrhiza* | Araceae | | Manganese transport protein mntH | |
| Spipo13G0011900 | SpNRAMP3 | *Spirodela polyrhiza* | Araceae | | natural resistance-associated macrophage protein1 | |
| Thecc1EG035168 | TcNRAMP1 | *Theobroma cacao* | Malvaceae | | Nramp transporter (71%U) | |
| Thecc1EG034751 | TcNRAMP2 | *Theobroma cacao* | Malvaceae | | NRAMP metal ion transporter 2 (77%T) | |
| Thecc1EG000729 | TcNRAMP3 | *Theobroma cacao* | Malvaceae | | Natural resistance-associated macrophage protein 3 (79%T) | |
| Thecc1EG035174 | TcNRAMP5 | *Theobroma cacao* | Malvaceae | | Nramp transporter (78%U) | |
| Thecc1EG027424 | TcNRAMP6 | *Theobroma cacao* | Malvaceae | | NRAMP metal ion transporter 6 (78%T) | |
| Vocar.0003s0226 | VcNRAMP1 | *Volvox carteri* | Volvocaceae | | PTHR11706:SF33 - metal transporter NRAMP1 | |
| Vocar.0008s0447 | VcNRAMP2 | *Volvox carteri* | Volvocaceae | | PTHR11706:SF8 - metal transporter NRAMP1 homolog | |
| GSVIVG01003954001 | VvNRAMP1 | *Vitis vinifera* | Vitaceae | | PTHR11706:SF38 - manganese transporter | |
| GSVIVG01008827001 | VvNRAMP2 | *Vitis vinifera* | Vitaceae | | PTHR11706:SF38 - manganese transporter | |
| GSVIVG01034237001 | VvNRAMP3 | *Vitis vinifera* | Vitaceae | | PTHR11706:SF33 - metal transporter NRAMP1 | |
| GSVIVG01008829001 | VvNRAMP4 | *Vitis vinifera* | Vitaceae | | PTHR11706:SF38 - manganese transporter | |
| GRMZM2G025680 | ZmNRAMP1 | *Zea mays* | Poaceae | | PTHR11706:SF46 - metal transporter NRAMP2-related | |
| GRMZM2G028036 | ZmNRAMP2 | *Zea mays* | Poaceae | | PTHR11706:SF46 - metal transporter NRAMP2-related | |
| GRMZM2G069198 | ZmNRAMP3 | *Zea mays* | Poaceae | | PTHR11706:SF33 - metal transporter NRAMP1 | |
| GRMZM2G168747 | ZmNRAMP4 | *Zea mays* | Poaceae | | PTHR11706:SF48 - manganese transporter | |
| GRMZM2G178190 | ZmNRAMP5 | *Zea mays* | Poaceae | | PTHR11706:SF51 - manganese transporter | |
| GAF08195 | PpMnhT | *Paenibacillus pini* | Paenibacillaceae | | Manganese transportern MntH | |

**Supplementary Table S3.** Characteristics of the primers used in expression analysis of cacao *TcNRAMP1, 2,* *3, 5* and *6* in this study.

| **Gene** | **Locus tag** | **Transcript ID** | **Primer sequence (5’-3’)** | **Amplicon size (bp)** |
| --- | --- | --- | --- | --- |
| *TcNRAMP1* | TCM_035168 | XM_007019080 | AGGAGCTTGTCTGCTTAGGG  GGCACACAGGTTACAAACGTA | 125 |
| *TcNRAMP2* | TCM_034751 | XM_007018502 | CCCGCAGGCTAAGTTGAGTT  ATTGTGTGGTCCCTTCTGGG | 141 |
| *TcNRAMP3* | TCM_000729 | XM_007047371 | GTCGCTGTGCTGGTGATAGT  AAAACCCCGGGAAACAAGGT | 147 |
| *TcNRAMP5* | TCM_035174 | XM_007019085 | GGAGCTTGTCTGCTTAGGGA  GGAAAGAGAGGTTACACATGCAAA | 125 |
| *TcNRAMP6* | TCM_027424 | XM_007023357 | GCCAATCTTTCAGAGCTAAGCC  CTGGTTTCCGGTACTAGCGA | 125 |
| *ACP1* | TCM_025966 | XM_007030091 | CAGCGAGAAAAGTGCCTAGA  AAATAAATAGACTTGAGTTCACAACAA | 127 |

**Supplementary Table S4.** List of primers used in cloning of cacao *NRAMP* genes in this study. The sequences of *attB* sites are underlined.

| **Gene** | **Purpose** | **Primer Name** | **Primer sequence (5’-3’)** |
| --- | --- | --- | --- |
| *TcNRAMP1* | Cloning into pCR™4Blunt-TOPO® vector | NRAMP1_F  NRAMP1_R | AACGTTGAGAAGAAAGGGAGAG  GGCACACAGGTTACAAACGTAAA |
|  | Construction of entry clone | NRAMP1/5_GTW_F  NRAMP1/5_GTW_R | GGGGACAAGTTTGTACAAAAAAGCAGGCTTCATGGGAAGTTTGCAGCAG  GGGGACCACTTTGTACAAGAAAGCTGGGTCCTACTCTGGCAGTGGTATGTCAGC |
| *TcNRAMP2* | Construction of entry clone | NRAMP2_GTW_F  NRAMP2_GTW_R | GGGGACAAGTTTGTACAAAAAAGCAGGCTTCATGAATTTCCTAAGCCGAGAC  GGGGACCACTTTGTACAAGAAAGCTGGGTCCTAACTTCCAGTGGCGGAAA |
| *TcNRAMP3* | Construction of entry clone | NRAMP3_GTW_F  NRAMP3_GTW_R | GGGGACAAGTTTGTACAAAAAAGCAGGCTTCATGCCGCCGGAAGAGA  GGGGACCACTTTGTACAAGAAAGCTGGGTCTCATTCAATTCCCTGTGTCTG |
| *TcNRAMP5* | Cloning into pCR™4Blunt-TOPO® vector | NRAMP5_F  NRAMP5_R | ATATAGTTGTTGTGAAGAAAGAGAGTG  GGAAAGAGAGGTTACACATGCA |
|  | Construction of entry clone | NRAMP1/5_GTW_F  NRAMP1/5_GTW_R | GGGGACAAGTTTGTACAAAAAAGCAGGCTTCATGGGAAGTTTGCAGCAG  GGGGACCACTTTGTACAAGAAAGCTGGGTCCTACTCTGGCAGTGGTATGTCAGC |
| *TcNRAMP6* | Construction of entry clone | NRAMP6_GTW_F  NRAMP6_GTW_R | GGGGACAAGTTTGTACAAAAAAGCAGGCTTCATGGCGGGCTCAAATTCTA  GGGGACCACTTTGTACAAGAAAGCTGGGTCTCAGTCCATGTCCTCCGTC |
| *TcNRAMP1, 2, 3, 5, 5s, 6* | Sequencing of insert in pCR™4Blunt-TOPO® vector/entry clone | M13_F  M13_R | TGTAAAACGACGGCCAGT  CAGGAAACAGCTATGACC |
| *TcNRAMP1, 2, 3, 5, 5s, 6* | Sequencing of insert in expression clone | pDR195GTW_P_F  pDR195GTW_T_R | CCAATTATGACCGGTGACG  GACAAGCCGACAACCTTGAT |
